# A comparative genomic analysis of lichen-forming fungi reveals new insights into fungal lifestyles

**DOI:** 10.1038/s41598-022-14340-5

**Published:** 2022-06-24

**Authors:** Hyeunjeong Song, Ki-Tae Kim, Sook-Young Park, Gir-Won Lee, Jaeyoung Choi, Jongbum Jeon, Kyeongchae Cheong, Gobong Choi, Jae-Seoun Hur, Yong-Hwan Lee

**Affiliations:** 1 Interdisciplinary Program in Agricultural Genomics, Seoul National University, Seoul, 08826 Korea; 2 Department of Agricultural Life Science, Sunchon National University, Suncheon, 57922 Korea; 3 Research Institute for Agriculture and Life Sciences, Seoul National University, Seoul, 08826 Korea; 4 Department of Agricultural Biotechnology, Seoul National University, Seoul, 08826 Korea; 5 Korean Lichen Research Institute, Sunchon National University, Suncheon, 57922 Korea; 6 Research Institute of Agriculture and Life Sciences, Seoul National University, Seoul, 08826 Korea; 7 Center for Fungal Genetic Resources, Seoul National University, Seoul, 08826 Korea; 8 Plant Immunity Research Center, Seoul National University, Seoul, 08826 Korea; 9 Present Address: SML Genetree Co. Ltd, Seoul, 08826 Korea; 10 Present Address: Smart Farm Research Center, Korea Institute of Science and Technology, Gangneung, 25451 Korea

**Keywords:** Fungal genomics, Fungi

## Abstract

Lichen-forming fungi are mutualistic symbionts of green algae or cyanobacteria. We report the comparative analysis of six genomes of lichen-forming fungi in classes Eurotiomycetes and Lecanoromycetes to identify genomic information related to their symbiotic lifestyle. The lichen-forming fungi exhibited genome reduction via the loss of dispensable genes encoding plant-cell-wall-degrading enzymes, sugar transporters, and transcription factors. The loss of these genes reflects the symbiotic biology of lichens, such as the absence of pectin in the algal cell wall and obtaining specific sugars from photosynthetic partners. The lichens also gained many lineage- and species-specific genes, including those encoding small secreted proteins. These genes are primarily induced during the early stage of lichen symbiosis, indicating their significant roles in the establishment of lichen symbiosis.Our findings provide comprehensive genomic information for six lichen-forming fungi and novel insights into lichen biology and the evolution of symbiosis.

## Introduction

Lichens exist in symbiosis, in which at least one fungus (mycobiont) lives in a mutually beneficial relationship with photosynthetic algae and/or cyanobacteria (photobiont)^1,2^. Since this dual nature was discovered by Schwendener ﻿in 1867^3^, numerous studies have demonstrated that basidiomycetes yeast^4–7^, as well as diverse microbiomes^8^, may cohabitate within lichen thalli. In lichen association, dominant fungal partners which produce basic morphological structure of lichens are determine the classification of lichens. The thallus structure composed of the fungal component retained the water for drought tolerance in extreme conditions^9,10^ as well as has a role as a shelter; protecting the photobionts from the external environment^2^. Moreover, the algal partner synthesizes carbohydrate products by photosynthesis and transfers this carbon source to the fungal partner to maintain the lichen association^11^.

Lichenization is common among fungi, with approximately 21% of fungal species forming lichens^2^. Lichenized symbiosis is not derived from a single phylogenetic clade^12^, but found in the Ascomycota classes Lecanoromycetes, Eurotiomycetes, Leotiomycetes, Dothideomycetes, and Arthoniomycetes, as well as in a few Basidiomycota classes^1,2^. Previous phylogenetic studies have suggested that lichenization evolved independently at least five times in distantly related lineages^12^. Such studies have also demonstrated that lichenization has been continuously maintained from the common ancestor of Lecanoromycetes, but was lost during the evolution of Lecanoromycetes. Due to this complex evolutionary history, many hypotheses have been proposed to account for the evolutionary time required for lichenization and its loss and re-evolution^13^.

To date, many studies have been conducted to elucidate the symbiotic nature of lichens. The successful re-association of lichen symbionts under laboratory conditions has facilitated microscopic observations of the fungal-algal interface during lichen establishment^14,15^. Thus, the early stages of lichenization, which ranges from ‘pro-contact’ to ‘growth together’, have been well investigated^15–17^; however, its late stages remain poorly understood. Although the aim of many studies is to identify symbiosis-related genes, until recently, we lacked the genetic transformation tools required to perform gene manipulation in lichen biology^18,19^. Thus, recent molecular studies have applied genetic transformation systems to elucidate lichen symbiosis.

However, the slow growth of several lichens and the difficulty of their culture in the laboratory have further required the development of genomic-level studies to gain an evolutionary understanding of lichen symbiosis. Genomics have advanced greatly since the sequencing of *Xanthoria parietina*^20–26^. Numerous studies have approached lichen symbiosis from a genomic perspective to identify evolutionary process of lichenization and symbiosis-related genes. *Endocarpon pusillum* was the first lichen to have been subjected to genomic analysis; early studies reported its symbiosis-related genes involved in nitrogen/sugar transport and metabolism with their expression during the re-synthesis stages^26^. Although continuous genomic studies investigating the key factors of lichen symbiosis^20,24,27^, recent descriptions of several additional genome sequences^28^, and the application of systems biology approach to lichen associations^29^ improve the knowledge of lichen symbiotic systems but determining how a symbiotic lifestyle evolved remains challenging. Mycorrhizal fungi, which are mutualistic symbionts associated with > 90% of land plants, have been studied extensively to identify their symbiotic nature. Large-scale genomic sequencing of mycorrhizal fungi has revealed that convergent evolution occurred via the loss of plant cell wall-degrading enzymes (PCWDEs) and the enrichment of transposable elements (TEs) and mycorrhiza-induced small secreted proteins (MiSSPs)^30–32^. Several molecular studies have also reported that secreted proteins play a crucial role in mycorrhizal symbiotic associations^33–35^.

In this study, we aim to conduct a comparative genomic analysis of four Lecanoromycetes species (*Gyalolechia flavorubescens*^36^, *Cladonia macilenta*^37^, *Cladonia metacorallifera*^38^, and *Umbilicaria muehlenbergii*^39^) and two Eurotiomycetes species (*Endocarpon pusillum* Z07020^26^ and R61883^40^) of lichen-forming fungi, which are evolutionary distant species. An additional 50 genomes from fungi with diverse lifestyles, including mycorrhizal fungi and close relatives of lichen-forming fungi, were also used to support the identification of the lichen-specific genomic features. We were going to perform a gene family gain/loss analyses in comparison with non-lichenized fungi to identify specific gene families of lichen-forming fungi. Finally, we re-synthesized *G. flavorubescens* and its algal partner *Trebouxia gelatinosa* and performed a time-series transcriptomic analysis of this re-synthesized lichen through RNA sequencing to reveal unique features of lichen symbiosis.

## Results

### Phylogenomic relationships and genomic similarity among lichen-forming fungi

**Figure 1:**
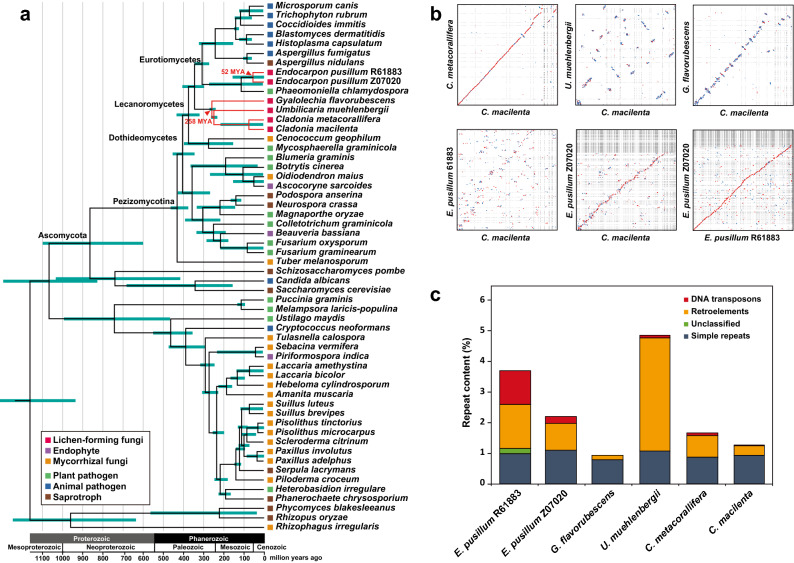
Phylogenomic and syntenic relationships among lichen-forming fungi and their repeat contents. (**A**) The phylogenomic tree shows that lichen-forming fungi are derived from many ancestors. Red branches indicate lichen-forming fungal lineages, and colored squares indicate lifestyle of a fungal species. The scale of the phylogenomic tree is millions of years, as calculated using the mcmctree function in the Phylogenetic Analysis by Maximum Likelihood software package. Blue error bars at each node indicate 95% highest posterior density (HPD) for node age. (**B**) Synteny dot plots of lichen-forming fungi. Red (blue) dots indicate forward (reverse) matches. (**C**) Repetitive sequence contents of lichen-forming fungi identified using RepeatMasker software. DNA transposons, retroelements, and unclassified repeats are classes of interspersed repeats.

The Lecanoromycetes and Eurotiomycetes lichen-forming fungi have similar genome sizes, ranging from 34.5 to 37.3 Mb, and similar numbers of genes (8294–9695) (Table 1). We conducted phylogenomic analysis of the six lichen-forming fungi including 50 fungal genome sequences (Supplementary Dataset S1). The phylogenomic tree showed that the four Lecanoromycetes species (*G. flavorubescens*, *C. macilenta*, *C. metacorallifera*, and *U. muehlenbergii*) and the two *E. pusillum* isolates were distantly related (Fig. 1A). This finding is consistent with previous reports that lichenization events evolved independently in multiple lineages^1,12,41^. The time-calibrated phylogeny suggests that Lecanoromycetes diverged approximately 258 million years ago from an ancestral fungus that may have been lichen-forming, and that divergence between *E. pusillum* isolates and the plant pathogen *Phaeomoniella chlamydospora* occurred approximately 52 million years ago.

**Table 1:** Genome statistics of the lichen-forming fungi.

Lichen species	Genome size (Mb)	Number of scaffolds	Number of genes	GC content (%)	References
*Endocarpon pusillum* R61883	37.13	59	9252	48.54%	Park et al., 2014
*Endocarpon pusillum* Z07020	37.33	308	9238	45.56%	Wang et al., 2014
*Gyalolechia flavorubescens*	34.47	36	9695	41.79%	Park et al., 2013
*Umbilicaria muehlenbergii*	34.81	7	8294	46.82%	Park et all., 2014
*Cladonia metacorallifera*	36.68	30	9030	43.81%	Park et al., 2014
*Cladonia macilenta*	37.12	240	8773	42.85%	Park et al., 2013

We analyzed the synteny of lichen-forming fungal genomes using *C. macilenta* as a reference. Dot plots revealed that both *Cladonia* species had a robust syntenic relationship, with several inverted blocks (Fig. 1B); however, as the evolutionary distance of species from *C. macilenta* increased, the syntenic relationship weakened. Because *C. macilenta* and *E. pusillum* belong to different classes, their syntenic relationship is nearly random, despite their both being lichen-forming fungi. The syntenic region of the two *Cladonia* species had 65.7–66.5% similarity, whereas the syntenic similarities between *C. macilenta* and other lichen-forming *G. flavorubescens* and *U. muehlenbergii* in Lecanoromycetes were 6.1–6.6% and 6.8–7.3%, respectively (Supplementary Table S1), and the two *Endocarpon* species in Eurotiomycetes had 3.4% and 3.5% similarity compared with *C. macilenta*.

Repetitive sequence content was also analyzed in lichen-forming fungi. The simple repeat content account for approximately 1% of all lichen-forming fungal genomes, but most DNA transposons were observed only in *E. pusillum* R61883, rather than other lichen-forming fungi (Fig. 1C). The portion of retroelements differ among lichen-forming fungi, Lecanoromycetes fungi (less than 1%), *E. pusillum* (1.5%), and *U. muehlenbergii* (4%). The total composition of repeat sequences in lichen-forming fungi was lower than that of other fungal species (Supplementary Fig. S1).

### Gene family expansion and contraction during the evolution of lichen-forming fungi

**Figure 2:**
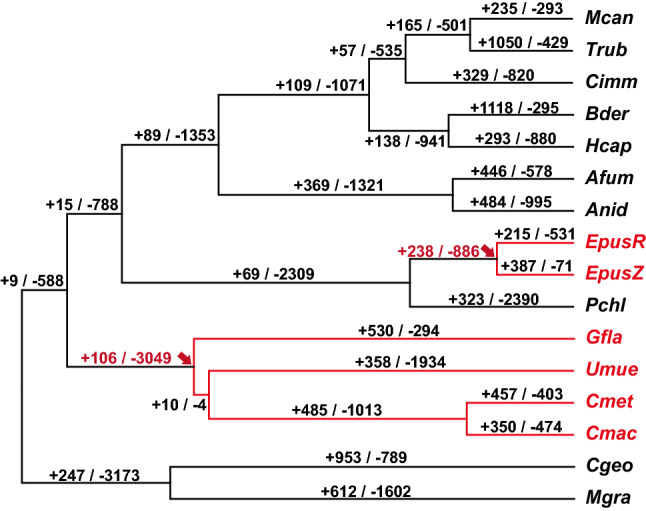
Gene family evolution in lichen-forming fungi and their relatives. Estimation of gene family expansion and contraction in lichen-forming fungi using the CAFE computational tool (P > 0.01). Red arrows indicate branch points where lichen-forming fungi diverged from non-lichenized ancestors. + and – indicate the numbers of expanded and contracted gene families, respectively. Only 16 species belonging to the Lecanoromyces, Eurotiomyces, and Dotidomyces closely related to lichen-forming fungi were shown, and the analysis results for all 56 species were placed in the Supplementary Figure S2. Species abbreviations: EpusR, *Endocarpon pusillum* R61883; EpusZ, *E. pusillum* Z07020; Gfla, *Gyalolechia flavorubescens*; Umue, *Umbilicaria muehlenbergii*; Cmet, *Cladonia metacorallifera*; Cmac, *Cladonia macilenta*. Abbreviations of other species names are provided in Supplementary Dataset S1.

Gene family expansion and contraction were analyzed based on orthologous genes across 56 fungal species including six lichen-forming fungi (Fig. 2). We estimated changes in gene family size when the two lichen-forming fungal clades Lecanoromycetes and *E. pusillum* diverged from different non-lichenized common ancestors. In Lecanoromycetes, 106 families expanded and 3049 contracted. Among the *E. pusillum* isolates, 238 families expanded and 886 contracted. Contractions were dominant in the lichen lineages, leading to a small total gene number in the lichen-forming fungi (Table 1; Supplementary Dataset S1). In both lichen-forming fungal clades, the cytochrome P450 (CYP) family expanded, whereas the glycoside hydrolase (GH), transcription factor (TF), and major facilitator superfamily (MFS) contracted (Table 2).

**Table 2:** Commonly expanded and contracted gene families in lineages of lichen-forming fungi.

Interpro ID	Term	Number of changed gene families in both lichen-forming fungi clades
**Expanded**		
IPR001128	Cytochrome P450	3
**Contracted**		
IPR013781	Glycoside hydrolase, catalytic domain	16
IPR007219	Transcription factor domain, fungi	12
IPR011701	Major facilitator superfamily	10
IPR001138	Zn(2)-C6 fungal-type DNA-binding domain	10
IPR005828	Major facilitator, sugar transporter-like	7

### Loss of plant cell wall degrading enzymes (PCWDEs) in lichen associations

**Figure 3:**
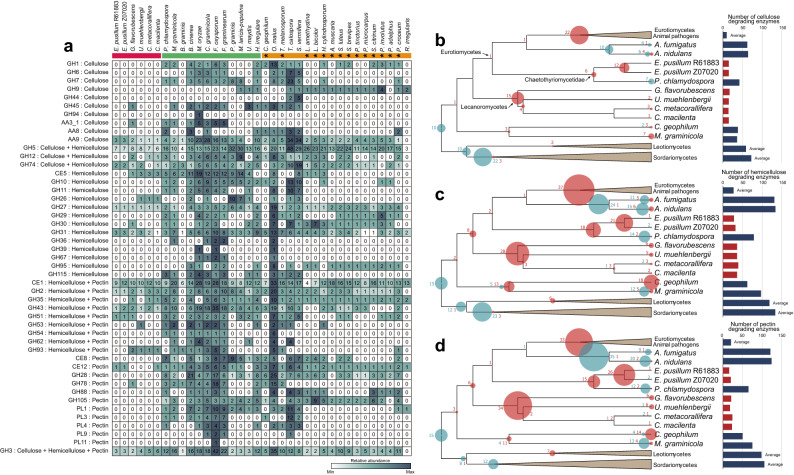
Loss of plant cell wall-degrading enzymes (PCWDEs) in lichen-forming fungi for association with algal partners. (**A**) Distribution of selected carbohydrate-active enzyme (CAZyme) families related to cellulose, hemicellulose, and pectin degradation among lifestyles. Red, green, and orange boxes indicate lichen-forming fungi, plant pathogens, and mycorrhizal fungi, respectively. Asterisk indicates ectomycorrhizal fungi, a class of mycorrhizal fungi. The distribution of PCWDEs in lichen-forming fungi was compared with plant pathogens known to have a large number of PCWDEs for pathogenicity and mycorrhizal fungi known to be associated with symbiosis formations. Analysis of other lifestyles is in Supplementary Dataset S2. CAZyme family abbreviations: GH, glycoside hydrolase; AA, auxiliary activities; CE, carbohydrate esterase; PL, polysaccharide lyase. Gene gain and loss analysis of (**B**) cellulose, (**C**) hemicellulose, and (**D**) pectin-degrading CAZyme families through species tree–gene tree reconciliation. Blue (red) circles indicate the number of gene gains (losses). Bubble size indicates the number of genes gained or lost. Bar graph indicates the total number of genes encoding PCWDEs in each species.

In both pathogens and symbionts, PCWDEs play essential roles in plant host cell wall remodeling for fungal colonization^42^. However, gene family expansion and contraction analysis (Fig. 3A) and the profiles of carbohydrate-active enzyme (CAZyme) genes (Supplementary Fig. S3) revealed a remarkable reduction of PCWDEs in six lichen-forming fungi. Plants and green algae have similar cell wall components, such as cellulose and hemicellulose, whereas pectin is unique to land plants and Charophycean green algae^43^. Lichen-forming fungi have fewer CAZyme genes involved in PCWDEs compared with plant-associated fungi, and a similar number compared with animal pathogens (Supplementary Fig. S3). Almost all polysaccharide lyase (PL) family genes, which act mainly in pectin degradation, have been lost in lichen-forming fungi. Only a few genes acting on cellulose (Auxiliary activity [AA] family 9, GH5, and GH3), hemicellulose (GH5, GH27, GH31, Carbohydrate esterase [CE] family 1, GH2, GH43, and GH3), and pectin (CE1, GH2, GH43, and GH3) are conserved in lichen-forming fungi (Fig. 3A). The number of PCWDE genes is dramatically decreased among lichen-forming fungi (Fig. 3A); decreasing patterns of PCWDE genes have been similarly observed in ectomycorrhizal fungi, which cannot penetrate host plant cell walls during colonization^30^.

We conducted gene tree–species tree reconciliation analysis to further infer the evolutionary relationships of PCWDE genes in lichen-forming fungi and their relatives (Fig. 3B–D). Lichen-forming fungi belonging to Lecanoromycetes lost 15 cellulose-degrading enzyme genes from their ancestral gene repertory (Fig. 3B). *E. pusillum* underwent two steps of gene loss: Chaetothyriomycetidae lost 6 genes, and then *Endocarpon* lost 12 cellulose-degrading enzyme genes. The plant pathogen *P. chlamydospora*, which is also a member of Chaetothyriomycetidae, regained several PCWDE genes subsequent to their loss in Chaetothyriomycetidae. The propensity of gene loss pattern in hemicellulose and pectin genes is similar to that of cellulose (Fig. 3C,D). The Eurotiomycetes lineage, which comprises only animal pathogens, also underwent massive loss of PCWDE genes whereas Leotiomycetes and Sordariomycetes, which include many plant-associated fungi, gained repertoires of PCWDEs for host invation. These results indicate that most genes related to the degradation of cellulose, hemicellulose, and pectin have been lost in lichen-forming fungi, but that these event occurred independently during the evolution of lichenization in different evolutionary lineages.

### Loss of sugar transporters during lichenization

**Figure 4:**
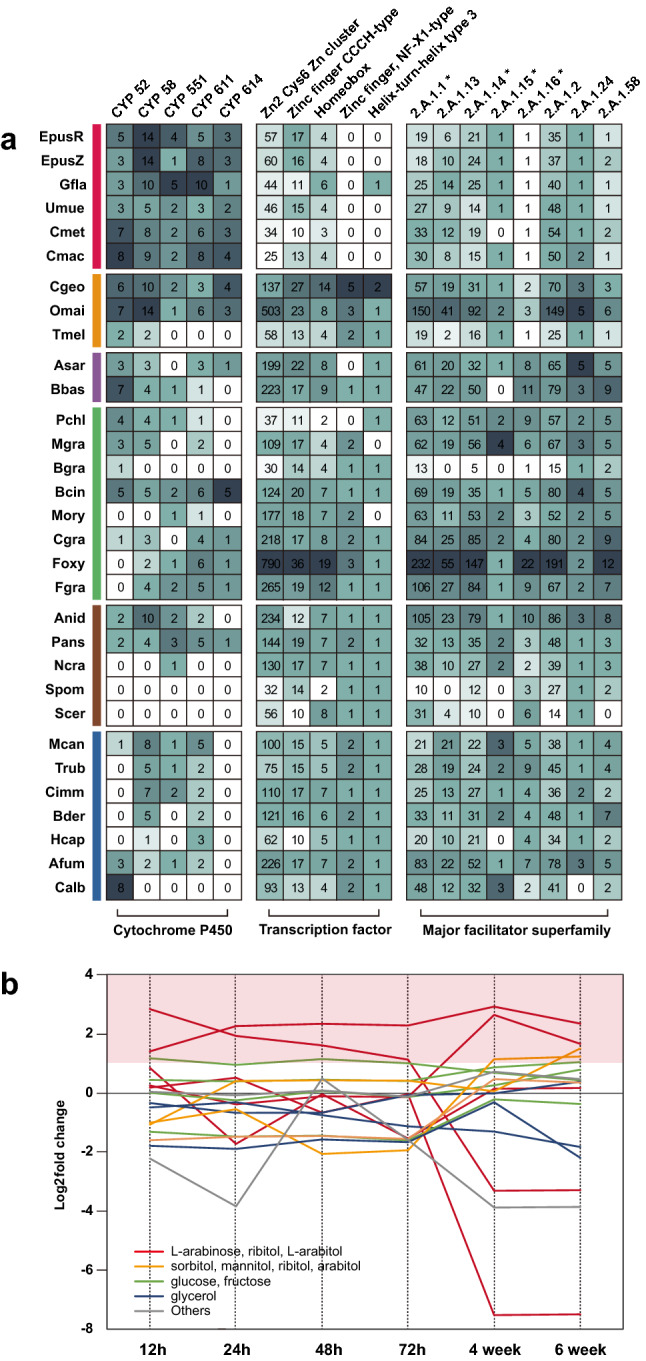
Distribution of expanded and contracted gene families in lichen-forming fungi. (**A**) Comparative analysis of selected families among the cytochrome P450 (CYP) and transcription factor (TF) families and major facilitator superfamily (MFS), which expanded or contracted in lichen-forming fungi. Colored boxes indicate fungal species lifestyles. Asterisk indicates rapidly contracted gene families. (**B**) Expression patterns of diverse polyol transporters in *G. flavorubescens*.

The MFS is the largest family of secondary transporters related to the movement of diverse solutes, especially sugar uptake^44^. However, MFS-type transporters underwent extensive contraction in six lichen-forming fungi, including the sugar porter (2.A.1), anion:cation symporter (2.A.1.14), aromatic acid:H + symporter (2.A.1.15), and siderophore-iron transporter (2.A.1.16) families (Fig. 4A). Because the type of sugar alcohols in symbiosis depends on the photosynthetic partners^45^, we further characterized the sugar transporters in lichen-forming fungi using dataset of *G. flavorubescens* expression during lichen resynthesis. A previous study defined 1 day post co-inoculation (PCI) as the ‘pre-contact’ stage of lichen fungi and algal partners, followed by 8 days PCI as the ‘contact’ stage, and 21 days PCI as the ‘growth together’ stage^15^. We measured gene expression during the early (12, 24, 48, and 72 h) and late (4 and 6 weeks) stages after lichen resynthesis. During resynthesis, the expression levels of four ribitol transporter genes were high at 4–6 weeks PCI, whereas those of other transporter genes were low (Fig. 4B). These findings are consistent with the reception of ribitol sugar alcohols by *G. flavorubescens* from its algal partner *Trebouxia* spp.^45,46^, and suggest that despite the extensive contraction of MFS-type transporters in lichen-forming fungi, these transporters may play important roles from 72 h to 4 weeks of lichenization.

### Massive contraction of transcription factor (TF) genes implies streamlined lichen-forming fungal genomes

**Figure 5:**
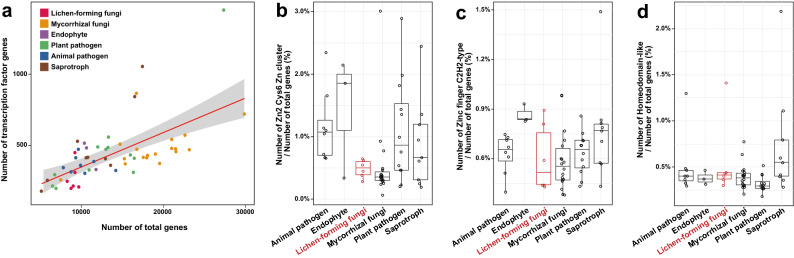
Major contributors of TF gene family contraction in lichen-forming fungi. (**A**) Correlation between the number of TF genes and the total number of genes. Red and grey lines are regression and error lines, respectively. Colored dots indicate fungal species lifestyles. (**B**–**D**) The three major TF families known to affect the total TF size. Red boxes indicate the distribution of lichen-forming fungi. (**B**) Zn2 Cys6 Zn cluster; (**C**) zinc finger C2H2-type; and (**D**) homeodomain-like.

Gene family expansion and contraction analysis revealed that the Zn2 cys6 Zn cluster DNA-binding domain, which is a fungal-specific TF family, was reduced in independent lineages of lichen-forming fungi (Table 2; Fig. 4A). We found that most other TF gene families had also contracted in lichen-forming fungi (Supplementary Fig. S4), particularly the zinc finger CCCH-type (IPR000571) and homeobox (IPR001356) families (Fig. 4A). The zinc finger, NF-X1 type (IPR000967), and helix-turn-helix type 3 (IPR001387) TF-type DNA-binding domains were not detected in the six lichen fungal genomes we analyzed. These losses in TF families led to the small number of TF genes in lichen-forming fungi compared with those of fungi with different lifestyles. Although the number of TF genes depends on the total number of proteins 40, lichen-forming and mycorrhizal fungi have fewer TF genes than expected (Fig. 5A). The Zn2 cys6 Zn cluster, zinc finger C2H2-type, and homeodomain-like DNA-binding domains are major contributors to the total number of TFs^47^; therefore we normalized these TF genes according to the total number of genes (Fig. 5B–D). However, only the Zn2 cys6 Zn cluster TF genes were responsible for the low number of TF genes in lichen-forming fungi, because percentage of zinc finger C2H2-type and homeodomain-like DNA binding domains were similar in lichen-forming fungi and in fungi with other lifestyles. Mycorrhizal fungi had similar distributions, suggesting that the reduction in TFs due to contraction of the Zn2 cys6 Zn cluster occurred in lichen-forming fungi and other symbionts.

Only *E. pusillum* R61883 underwent possible duplication in specific TF families, including homeodomain-like (IPR009057) and helix-turn-helix psq (IPR007889) families (Supplementary Fig. S4 and S5A). We hypothesize that transposons and transposition of DNA-mediated genes (GO:0006313) particularly abundant in this sample may have caused these duplications (Supplementary Fig. S5A). Because DNA transposons were near (3 kb) the duplicated homeodomain-like and helix-turn-helix psq families, we hypothesize that the expansion of TF families was influenced by repeat elements (Fig. 4B).

### Expanded cytochrome P450 (CYP) genes and secondary metabolites involved in lichen symbiosis

CYPs are heme-containing monooxygenases involved in a variety of metabolic processes^48^. Genes in the CYP52, CYP58, CYP551, CYP611, and CYP614 families are more numerous in lichen-forming fungi than in other analyzed genomes (Fig. 4A). Expanded CYP genes in lichen-forming fungi are separated from those of fungi with other lifestyles, indicating that this feature evolved uniquely from other fungi. CYP52 and CYP58 are involved in n-alkane and fatty acid assimilation and trichothecene biosynthesis^49^. The CYP551, CYP611, and CYP614 families have not been characterized, but may be involved in the symbiotic lifestyle because most of these CYP genes are lichen genes (Fig. 4A; Supplementary Fig. S6).

Lichen-forming fungi synthesize various unique secondary metabolites^2^. We found more polyketide synthase (PKS) genes in lichen-forming fungi, mainly in Lecanoromycetes, than in 2). Reconciliation analysis revealed that the gain of these PKS genes occurred after lichen-forming fungi emerged from non-lichenized ancestors (Supplementary Fig. S7). Although the *E. pusillum* isolates are distantly related to Lecanoromycetes lichens, lichen-forming fungi shared many PKS genes in the phylogenetic analysis (Supplementary Fig. S8); we identified a lichen-specific PKS group that consisted entirely of lichen species, except *G. flavorubescens*. Sequence similarity analysis based on a blast search revealed that this is a lichen-specific PKS gene, with no ortholog in other fungal species (Supplementary Fig. S9A). Instead, *G. flavorubescens* have species-specific PKS genes (Fig. 7B). This genomic evidence is consistent with previous findings of the presence of unique secondary metabolites synthesized in lichen-forming fungi^50–52^.

Transcriptomic data for the resynthesis of *G. flavorubescens* and *T. gelatinosa* were used to investigate the relationships among two expanded gene families (PKS and CYP) and lichen symbiosis. Several PKS and CYP genes were highly expressed only during the early stage (at 12, 24, 48, and 72 h), whereas other genes were induced only during the late stage (4 and 6 weeks) (Supplementary Fig. S10). All lichen-specific PKS genes were induced only during the early stage of symbiosis (Supplementary Fig. S10B). The expanded gene families appear to be involved in producing various compounds and secondary metabolites, as previously described^2,50–52^. However, the expression patterns of lichen-specific PKS genes indicate that the lichen-specific PKS products are induced during the early stage of symbiosis.

### Lichen-specific genes of six lichen-forming fungi

In addition to the loss of unnecessary genes in lichen-forming fungi, we attempted to identify newly gained genes that may contribute to their unique symbiotic lifestyle. Ortholog clustering analysis of the six lichen-forming fungi identified 3051 core groups, whereas clustering with only four Lecanoromycetes identified 3,468 core groups (Supplementary Fig. S11A and B; Supplementary Table S3). Thus, the number of core gene clusters in lichen-forming fungi remained consistent regardless of the lichen-forming fungi in different classes.

We identified 5498 lichen-specific orthogroups, including species-specific genes, after clustering with an additional 50 fungal genomes. The number of core groups was substantially reduced among lichen-forming fungi, leaving only one lichen-specific core group (Supplementary Fig. S11C and D). This finding suggests that no universal lichen-forming fungal gene sets are involved in their symbiosis and that, rather than core genes, they have many genus- or species-specific genes (Supplementary Table S3), which are also important in mycorrhizal symbiosis^30^.

Lichen-specific genes were functionally annotated through gene ontology (GO) analysis using biological process terms. GO terms revealed no association with approximately 90% of the genes in *E. pusillum* R61883, 97% in *E. pusillum* Z07020, 99% in *G. flavorubescens*, 99% in *U. muehlenbergii*, 88% in *C. metacorallifera*, and 89% in *C. macilenta* (Supplementary Fig. S12A); therefore, these genes were likely newly gained during the evolution of lichen symbiosis. Common function of the functionally annotated genes in the six lichen-forming fungi included oxidation–reduction processes, protein phosphorylation, transmembrane transport, carbohydrate metabolic processes, and transcription regulation (Supplementary Fig. S12B). However, genes involved in DNA-mediated transposition were found only in *Endocarpon* species, primarily in *E. pusillum* R61883. This difference appears to be related to the abundance of DNA transposons and the expansion of specific TF families in *E. pusillum* R61883, as mentioned above (Supplementary Fig. S5).

### Symbiosis-induced genes in *G. flavorubescens*

**Figure 6:**
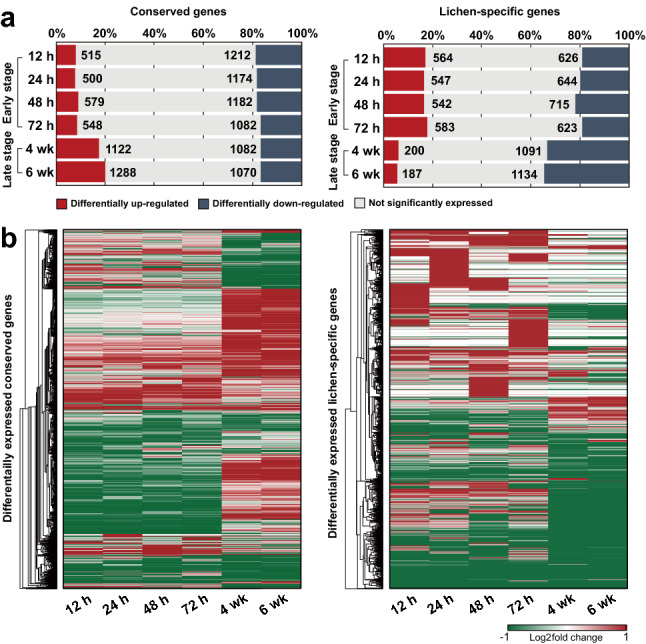
Symbiosis-induced genes in *G. flavorubescens.* Gene expression profiles of conserved and lichen-specific genes in *G. flavorubescens*. (**A**) Differentially up- (log_2_ fold change > 1) and downregulated (log_2_ fold change ≤  − 1) genes at each time stage. Gray bars indicate genes that were not significantly expressed. (**B**) Expression patterns of lichen-specific and conserved genes.

Genome-wide expression profiling was performed using *G. flavorubescens* and its algal partner *T. gelatinosa* to determine the possible roles of lichen-specific genes and conserved genes during lichenization. Of the conserved genes with orthologs in non-lichenized fungi, 17–20% were upregulated 4–6 weeks PCI, whereas 8–9% were upregulated at 12–72 h PCI (log_2_ fold-change > 1) (Fig. 6A). Most genes that were upregulated during the late-stage were not affected during the early stage (Fig. 6B), indicating that different conserved genes are associated with the early and late stages. Lichen-specific genes exhibited a different pattern from conserved genes, with more genes upregulated during the early stage (17–18%) than the late stage (6%) (Fig. 6A). Similar to conserved genes, different genes were upregulated during the early and late stages, suggesting that lichen-specific genes that are highly induced during the early stage are no longer necessary during the late stage. Different lichen-specific upregulated genes were involved at each time point, even within each stage (Fig. 6B).

GO enrichment analysis was performed on functionally annotated conserved genes that were differentially expressed during the early and late stages (Supplementary Table S4). Genes that were up- or downregulated only during the early or late stage were defined as differentially expressed. Genes that were differentially upregulated during the early stage were significantly enriched in terms of epigenetic mechanisms, including chromosome organization (GO:0051276), DNA repair (GO:0006281), peptidyl-amino acid modification (GO:0018193), protein acylation (GO:0043543), and histone modification (GO:0016570). In contrast, terms related to glucose (GO:0006,096 and GO:0009070) and lipid (GO:0042157, GO:0042158, and GO:1903509) metabolism were significantly enriched during the late stage, suggesting that the early and late stages play different roles in lichen symbiosis.

Glucose metabolism is important in lichen-forming fungi, which absorb photosynthetic products from their algal partners and convert them into glucose or fructose for fungal metabolism^26^. Glycolysis/gluconeogenesis pathway analysis of the differentially expressed genes mapped only genes induced during the late stage to the pathways (Supplementary Fig. S13), indicating that conversion of the obtained monosaccharides into energy sources occurs actively during the late stage.

### Small secreted proteins (SSPs) in lichen-forming fungi are involved in establishment and maintain the symbiosis

**Figure 7:**
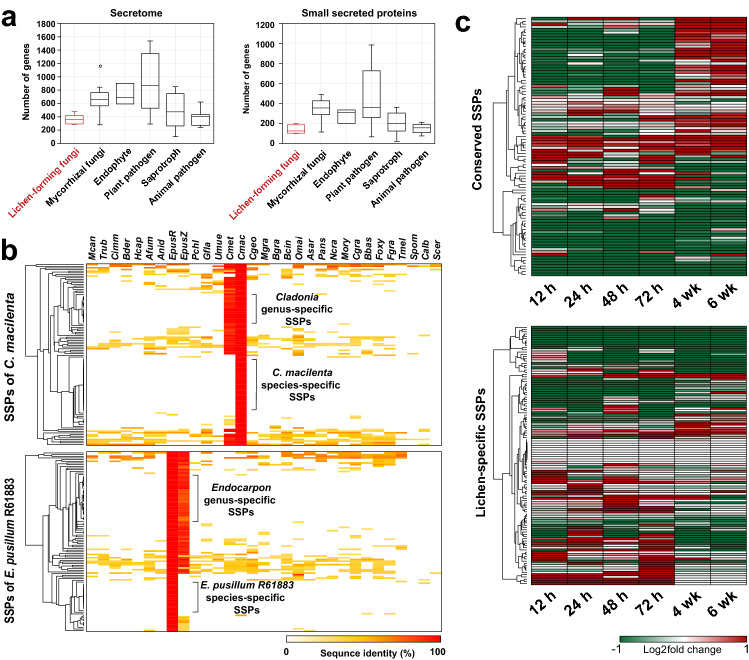
Lichen-specific small secreted proteins (SSPs) from *G. flavorubescens* induced in early lichen symbiosis. (**A**) Box plot of the number of secretomes and SSP distributions in lichen-forming fungi compared with other lifestyles. (**B**) Lichen-specific SSPs in *C. macilenta* and *E. pusillum* R61883. Ortholog SSPs of 56 fungal species were identified using blast and SSPs *of C. macilenta* and *E. pusillum* R61883 as references (E = 1 × 10^−5^). Abbreviations for fungal species are provided in Supplementary Dataset S1. (**C**) SSP expression in *G. flavorubescens* was classified as conserved (top) or lichen-specific (bottom).

Although small secreted proteins (SSPs) are virulence factors in pathogenic fungi and are important for symbiosis in mycorrhizal fungi^53^, their roles in lichen-forming fungi remain unclear. We found that lichen-forming fungi had 286–482 secreted proteins and 107–207 SSPs (Supplementary Fig. S14); these numbers were smaller than those for other fungi with different lifestyles, especially plant-associated symbionts (Fig. 7A). Most SSPs found in lichen-forming fungi were genus- or species-specific according to the blast results (Fig. 7B; Supplementary Fig. S15). Sequence identity analysis of SSPs in *C. macilenta* revealed that 32% of the SSPs were *Cladonia*-specific and 39% were species-specific, whereas 43% of SSPs in *E. pusillum* R61883 were *Endocarpon*-specific and 31% were species-specific (Fig. 7B). In contrast, 76% of SSPs in *G. flavorubescens* and 75% in *U. muehlenbergii* were species-specific, as both species lacked closely related species, in our dataset (Supplementary Fig. S15). Although both of these lichen-forming fungal species belong to Lecanoromycetes, and both have *Trebouxia* spp. as algal partners, their SSPs differed markedly based on sequence similarity, suggesting that SSPs of lichen-forming fungi may not just dependent on their photobiont, but have been independently gained during speciation.

Like all genes, SSPs in *G. flavorubescens* had different expression profiles for conserved or specific genes. Most conserved SSPs were highly upregulated during the late stage of resynthesis, although some were also constitutively expressed during the early stages (Fig. 7C). However, most genus- and species-specific lichen SSPs were upregulated at different time points during the early stage.

## Discussion

The main goal of symbiosis research is to determine how the beneficial associations evolved and to identify genes involved in the establishment and functioning of symbioses. However, to date, these features have been only partially investigated in lichen-forming fungi. All that is known about the evolution of lichen symbionts independently from non-lichenized ancestors was acquired from studies of small subunit and large subunit rDNA markers^12,41^. We used whole-genome sequences to determine phylogenetic relationships more accurately. Our comparative analysis revealed that the lichen-forming fungi experienced massive reductions in unnecessary genes during symbiosis with their algal partners. Newly acquired lineage- and species-specific genes are involved in establishing lichen symbiosis, whereas conserved genes maintain the relationship.

PCWDEs are involved in host cell wall remodeling in mycorrhizal symbiosis^54^ and confer virulence to fungal plant pathogens^55^. However, we found that lichen-forming fungi experienced large contractions in PCWDE genes compared with their non-symbiotic ancestors. Pectin-degrading enzymes may no longer be necessary for algal host association in lichen symbiosis, because this cell wall component is unique to Charophyceae algae and land plants, but is not present in Chlorophyte green algae, which can form lichen^43^. Consequently, most pectin-degrading enzyme genes have been lost in lichen-forming fungi derived from non-lichenized fungi. Nevertheless, cellulose and hemicellulose enzyme genes underwent contractions similar to pectin, although they are common cell wall components in both plants and green algae^43^. Lichen-forming fungi have simple wall-to-wall apposition or develop highly differentiated non-breaking symbiotic structures called intraparietal haustoria, as well as intracellular haustoria, which penetrate algal cell walls^56^. Although the haustoria of the lichen species used in this study were not observed directly, we suggest that they do not penetrate the host algal cell walls for colonization, as proposed in previous studies^56–58^. These symbiotic forms may lead to the non-functionalization of PCWDEs, which leads to gene loss^59^. However, a recent study using a vast number of lichen-forming fungal genomes revealed that not all lichen-forming fungi lost large numbers of PCWDE genes^28^. Taking this findings into consideration, the overall trend of our data suggests the loss of PCWDE genes in lichen, with several exceptional cases. Ectomycorrhizal fungi have similar symbiotic relationships with their hosts and have lost many PCWDE genes, unlike endomycorrhizal fungi and plant pathogens^30,32,60^. Their symbiotic structure also does not penetrate the plant cell walls^54,61^; which suggests that the loss of the ability to degrade cell walls in lichen-forming and mycorrhizal fungi is a consequence of their symbiotic fungus–host interface.

Many MFS-type transporters were also lost, although carbohydrate movement from the algal partner to the lichen-forming fungus is important in lichen symbiosis^62^. Although previous lichen genomic studies have also reported reductions in sugar transporters^20,26^, we found that these losses were common in lichen lineages, not only in each species. Lichen-forming fungi likely use specific transporters, as they receive different mobile carbohydrates, such as ribitol, sorbitol, and glucose, from their algal partners^45^. We propose that the extensive loss of sugar transporter genes is a result of the dispensability of common sugar transporters. The upregulation of ribitol transporter genes during the late stage of *G. flavorubescens* symbiosis supports this hypothesis, whereas other sugar transporters exhibited no significant changes. Algal partners export carbohydrates only during symbiosis^62^, such that the completion of lichen symbiosis and the initiation of nutrient exchange occur between 72 h and 4 weeks PCI.

As a result of contractions in diverse gene families (e.g., PCWDEs, sugar transporters, and TFs), the genome size and total number of genes of lichen-forming fungi are lower than those of other fungal species, especially plant-associated fungi. This is unsurprising because gene losses are widespread among all organisms^59^, and genome reduction is a dominant evolutionary process resulting in the loss of non-functionalized genes^63^. Symbionts have significantly reduced genomes due to their dependence on photosynthetic partners^64,65^. The loss of energy-production genes in the mitochondrial genomes of lichen-forming fungi is an example of this reductive evolution^65^. Although to date genome streamlining has been evaluated only in bacterial and mitochondrial genomes, we suggest that it can also occur in the nuclear genome. The loss of TF genes is another consequence of this evolutionary mechanism, and the loss of many genes and dependency on the host may influence the size of TF families.

Both massive gene losses and independent gene gains have occurred in lichen-forming fungi. Each species has many unique genes. In this study, we attempted to identify lichen-specific core genes, but found only one orthogroup. Similarly, mycorrhizal fungi lack universal symbiosis genes^60^; instead, newly gained lichen-specific genes, including lineage- and species-specific genes, appear to be more related to their lifestyles, with high expression during early symbiosis, when they influence their partners^14^. Most SSPs were also genus- or species-specific and had similar expression patterns. The transient expression of each set of specific genes suggests that they are activated differently during each period of the early stage. Based on the sequential expression of effector proteins in the plant pathogen *Colletotrichum higginsianum*, different SSPs may be involved during each stage of host–pathogen interaction^66^. Because the functions of most lichen-specific genes are unknown, and the SSPs of symbionts play essential roles in maintaining mycorrhizal symbiotic relationships^33,34^, lineage- and species-specific genes may play significant roles in the establishment of lichen symbiosis. The functionally annotated conserved genes that may be involved in maintaining their symbiotic relationships were induced mainly during the late stage of lichen symbiosis, when recognition and contact with the partner are completed, growth is continued^15^, and metabolic processes such as nutrient exchange are activated. For example, genes involved in glycolysis are expressed differentially during the late stage of lichen symbiosis, allowing the use of sugars obtained from photosynthetic partners. These findings indicate that lichen-specific genes and conserved genes play roles in different stages of lichen resynthesis.

The evolutionary pattern of gene loss of lichen-forming fungi is similar to that of ectomycorrhizal fungi. Both symbionts lost the ability to degrade cell walls and gained lineage-specific genes that may be involved in symbiosis; this evolutionary process is well known in mycorrhizal fungi^30,32^. However, ectomycorrhizal fungi still retain PCWDEs, including GH28, GH88, CE8, and GH30, which are induced in mycorrhizal symbiosis for host cell wall modification^30^, whereas lichen-forming fungi have lost most of these genes. The number of effector proteins remains unknown^67^. However, because lichen-forming fungi have fewer SSPs than mycorrhizal fungi or other plant-associated fungi, we suggest that the number of SSPs depends on the complexity of their host. Because green algae are the ancestors of land plants and have evolved to become more complex in terms of cellular organization^68^, lichen-forming fungi may not require many SSPs to interact with the defense mechanism of their living host. Most gene family expansion occurred during the speciation of each mycorrhizal fungus (Supplementary Fig. S2), which suggests that lichen-forming fungi and mycorrhizal fungi underwent different unknown evolutionary processes to develop their lifestyles.

This study is the first comparative analysis of diverse lichen-forming fungi using whole genomes to clarify elements of lichen symbiosis. We found that the loss of non-essential genes, such as specific families of PCWDEs, sugar transporters, and TFs, streamlined the genomes of lichen-forming fungi, providing new insights on lichen symbiosis. Lineage- and species-specific genes, including SSPs, play a role during the early stage of lichen symbiosis, and may be involved in recognition between lichen-forming fungi and their partners. These findings advance understating of the evolution of symbiotic lifestyles and the determinants contributing to lichen symbiosis. Genomic resources may contribute to future molecular functional studies of the unrevealed biological functions of significant factors in lichen symbiosis.

## Materials and methods

### Genome resources of fungal species and ortholog clustering

We sequenced genome of five lichen-forming fungi including *G. flavorubescens* KoLRI002931 (accession no. AUPK01000000)^36^, *C. macilenta* KoLRI003786 (AUPP01000000)^37^, *C. metacorallifera* KoLRI002260 (AXCT02000000)^38^, *U. muehlenbergii* KoLRILF000956 (JFDN01000000)^39^ and *E. pusillum* R61883 (JFDM01000000)^40^. *E. pusillum* Z07020 (APWS00000000)^26^ in previous researches and the other 50 fungal genomes used for comparative analysis were downloaded from Broad Institute (http://www.broadinstitute.org/), JGI fungal genome portal MycoCosm (http://jgi.doe.gov/fungi) and NCBI-GenBank database. The predicted protein sequences from 56 fungal genomes were clustered by OrthoFinder v2.2.7 with the default program settings^69^. The conserved and lichen-specific genes were annotated by GO term annotation using InterProScan version 60^70^.

### Phylogenetic analysis and divergence time estimation

Total proteins of 56 fungal genomes were used to construct a whole genome-based phylogenomic tree using CVtree3 with k-tuple 7^71^. Initial curation of the divergence time for the major fungal taxa was achieved by Timetree^72^, and the divergence times were estimated by MCMCtree in PAML package version 4.8^73^ using molecular markers, including actin (ACT1), translation elongation factor EF1-α (TEF1), RNA polymerase II large subunits (RPB1 and RPB2) and β-tubulins (TUB1 and TUB2). The final phylogenomic tree with divergence times was visualized by MEGA version 7.0.26^74^.

### Repetitive sequence and whole genome synteny analysis

The repeat contents were analyzed using TRF and rmBlastN, parts of RepeatMasker v4.0.5 package with RepBase 21.05 fungi library^75^. For pairwise genomic comparisons, MUMmer v3.23^76^ was used for aligning and comparing the whole genomes between lichen-forming fungi and the other fungal genomes.

### Gene family evolution analysis and gene family annotation

CAFE (Computational analysis of gene family evolution) v2 was used to find out the gene families with significant changes in size (P < 0.01)^77^. The time-calibrated phylogenetic tree and gene families identified by ortholog clustering were used for this analysis. Functional annotations of expanded and contracted gene families were identified by domain-based InterProScan v60^70^. The cytochrome P450 genes were firstly identified with Fungal Cytochrome P450 Database (FCPD)^48^, and then BLAST analysis against the P450 database in David Nelson cytochrome P450 web site^78^ for nomenclature. The secondary metabolite biosynthesis gene clusters, including PKS, NRPS, and DMATs were identified by SMURF^79^. Candidate MFS transporters were obtained using the Transporter Classification Database (TCDB)^80^. Diverse polyol and monosaccharide transporters of *G. flavorubescens* were predicted by BLAST search (identity > 30, query coverage > 60) with functionally characterized transporter genes listed in^81^. The transcription factors were predicted by InterProScan v60 using the previously annotated DNA-binding domains^47^, and PCWDE encoding CAZyme families were annotated by HMMER search against dbCAN CAZyme domain HMM database^82^. The secretomes and SSPs were predicted by the method described previously^53^. The maximum-likelihood phylogenetic trees of CYP, PKS, PCWDEs, homeodomain-like and helix-turn-helix psq TF genes were constructed using RAxML version 8.2.9 with a bootstrap value of 1000^83^. Aligning protein sequences using ClustalW 2.1^84^ and remove poorly aligned regions by trimAl v1.2^85^ were preceded before phylogenetic analysis. We reconciled the gene trees of PCWDEs resulting from this analysis with the species tree using NOTUNG 2.9^86^.

### RNA extraction and expression analysis

Actively growing *G. flavorubescens* KoLRI002931 mycelia were collected and macerated the mycelia into 10 mL of sterilized distilled water using a homogenizer (Ika, T10 basic, German). The macerated mycelia were dropped on malt extract agar medium (Difco), incubated at 15 ° for 4–6 weeks, and then covered 50 μL of 2 weeks old *Trebouxia gelatinosa* cell suspension (1 × 10^8^/mL) which is partner alga of *G. flavorubescens*. The plates were incubated at 15 °C without light. For harvesting samples at different time points during re-synthesis between *G. flavorubescens* and *T. gelatinosa*, all samples were collected 0 h, 12 h, 24 h, 48 h, 72 h, 4 weeks, and 6 weeks after re-synthesis, immediately frozen using liquid nitrogen, and stored at − 80 ° until processed. The whole samples on the medium were collected from three replicates of three biological repeats except 4 and 6 weeks. Total RNA was extracted using an Easy-Spin Total RNA Extraction Kit (iNtRON Biotechnology, Seoul, Korea). RNA sequencing was performed at Macrogen Inc. (Seoul, Korea) using Illumina HiSeq platform.

The NGS QC Toolkit ver. 2.3.3^87^ was used to remove adaptors, low-quality sequences and sequences containing more than 5% N to obtain clean reads. Since the sequences of *G. flavorubescens* and *T. gelatinosa*, the partner alga, were mixed in the clean reads, the algal reads were eliminated using BWA (0.7.9a-r786)^88^. Then the paired-end clean reads were aligned to *G. flavorubescens* genome using TopHat v2.0.12^89^ and the gene expression levels were calculated as FPKM (Fragment Per Kilobase of transcript per Million mapped reads) using cufflink v.2.2.1^90^ and cuffdiff v.2.21^91^. The FPKM value of 0 h, 12 h, 24 h, 48 h, and 72 h PCI were calculated with three biological repeats, and 4 weeks and 6 weeks PCI were calculated without repeats. Fold changes were calculated simply using a modified function, log2([FPKM_SYMBIOSIS_ + 1]/[FPKM_MYCELIA_ + 1]). The hieratical clustering of protein expressions in the heatmaps was performed using the Euclidean clustering distance by Morpheus run by Broad institute (https://software.broadinstitute.org/morpheus). Significantly enriched GO terms in symbiosis induced genes were identified using R package topGO version 2.38.1 with threshold p < 0.05^92^. KEGG pathway mapping analysis using differentially expressed genes was performed by KEGG Automatic Annotation Server (KAAS) web sites^93^.

## Supplementary Information

Supplementary Information 1.

Supplementary Information 2.

Supplementary Information 3.

## Data Availability

The transcriptome sequence data of *G. flavorubescens* have been deposited in the NCBI Sequenced Read Archive (SRA) under accession number PRJNA210248.
