# Supplementary material for: A comparative genomic analysis of lichen-forming fungi reveals new insights into fungal lifestyles
Source: Sci Rep. 2022 Jun 24;12:10724. doi: 10.1038/s41598-022-14340-5 (PMC9232553; doi:10.1038/s41598-022-14340-5)
Supplement: Supplementary file 3 — Supplementary Information 3. [file 41598_2022_14340_MOESM3_ESM.docx]

Supplementary Information

# Supplementary Note

**Delineation of the two *E. pusillum* strains as different species by comparative genomics**

*E. pusillum* strains Z07020 and R61883 have been described as the same species in previous studies because they are morphologically indistinguishable^1,2^. However, morphological classification is insufficient for species delineation, and molecular genetics has been used to solve this problem^3,4^. To investigate this possibility, we compared the conserved proteins (ACT1, TEF1, TUB1, and TUB2) and the whole genomes of the two *E. pusillum* strains. Genomes of other species in the same genus and other strains in the same species were used for comparison. The sequences of conserved proteins among strains in the same species were identical, whereas the proteins of different species in the same genus had differences (Supplementary Table S5). Comparison between the two *E. pusillum* strains showed that TEF1 and TUB2 genes contained mismatches, similar to the results for different species in the same genus. Whole-genome synteny analysis also showed that the *E. pusillum* strains had a relationship similar to fungi of different species in the same genus (Supplementary Fig. S16 and Supplementary Table S6). Our results show that their genomic synteny and repeat contents differ significantly. This difference provides decisive evidence that their genomes have evolved into different species. Therefore, we determined that the two *E. pusillum* strains belong to different species within the same genus. Moreover, some gene families such as TFs expanded exclusively in *E. pusillum* R61883. This finding suggests that repetitive sequences, including transposable elements, can induce changes in genome structure that lead to speciation^5,6^ and may have driven the rapid evolution of *E. pusillum* R61883.

# Supplementary Figures and Tables


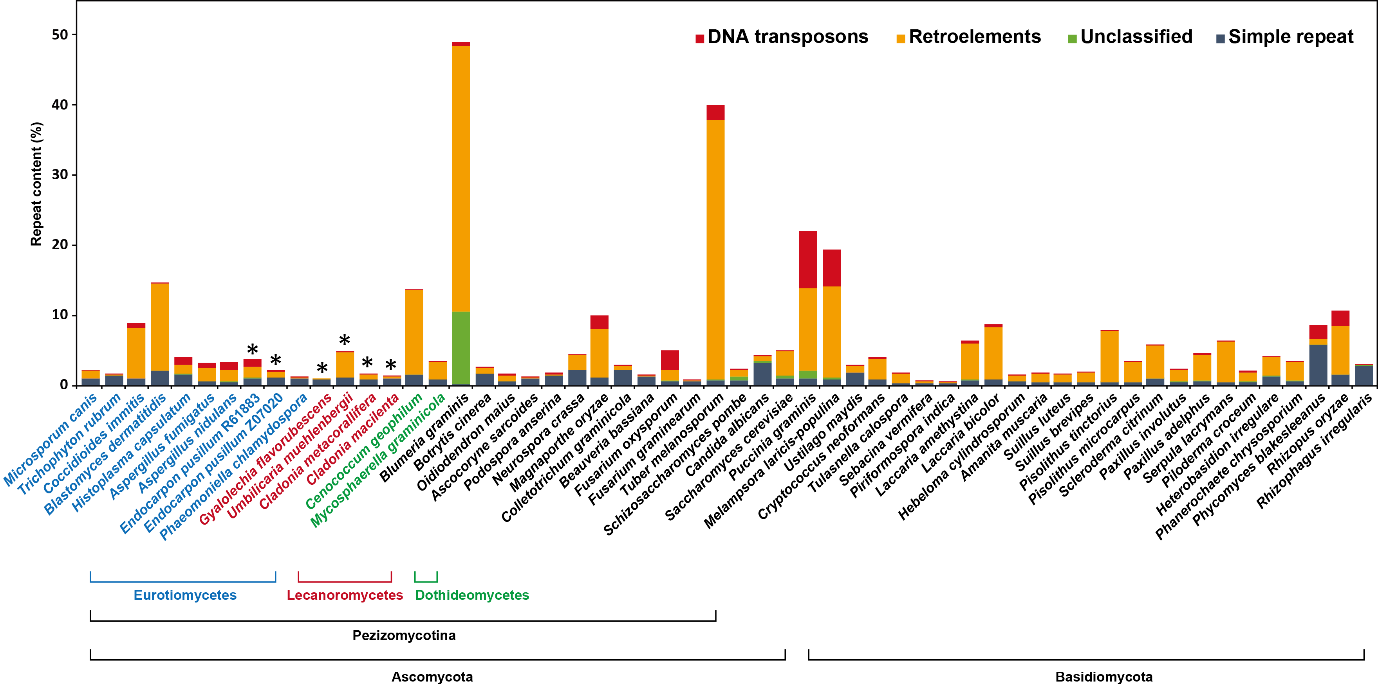


**Supplementary Fig. S1. Repeat contents of 56 fungal species**

Repetitive sequences of 56 fungal species analyzed by RepeatMasker (Smit et al., 2013). Each colored bars represent a class of repeats. The species name in blue represents Eurotiomycetes, red represents Lecanoromycetes, and green represents Dothideomycetes. The bar graphs with an asterisk indicate the lichen-forming fungi.


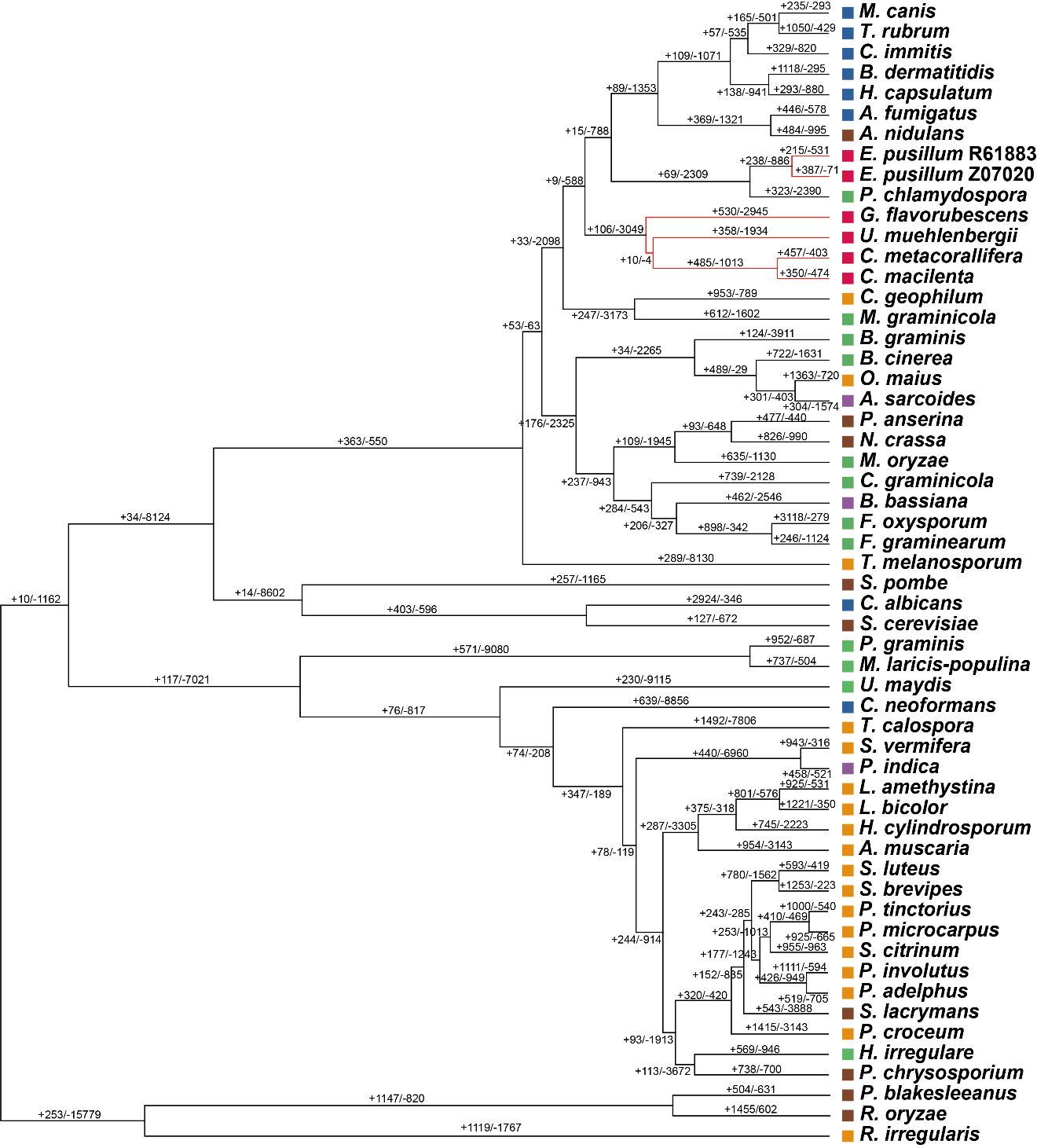


**Supplementary Fig. S2. Gene family evolution in 56 fungal species**

Expanded and contracted gene families across the 56 fungal species which have diverse lifestyles. Estimation of changed gene families was performed by CAFE (P > 0.01). Red branches represent the divergence of lichen-forming fungi from non-lichenized ancestors. + and – indicate the respective numbers of expanded and contracted gene families, respectively.


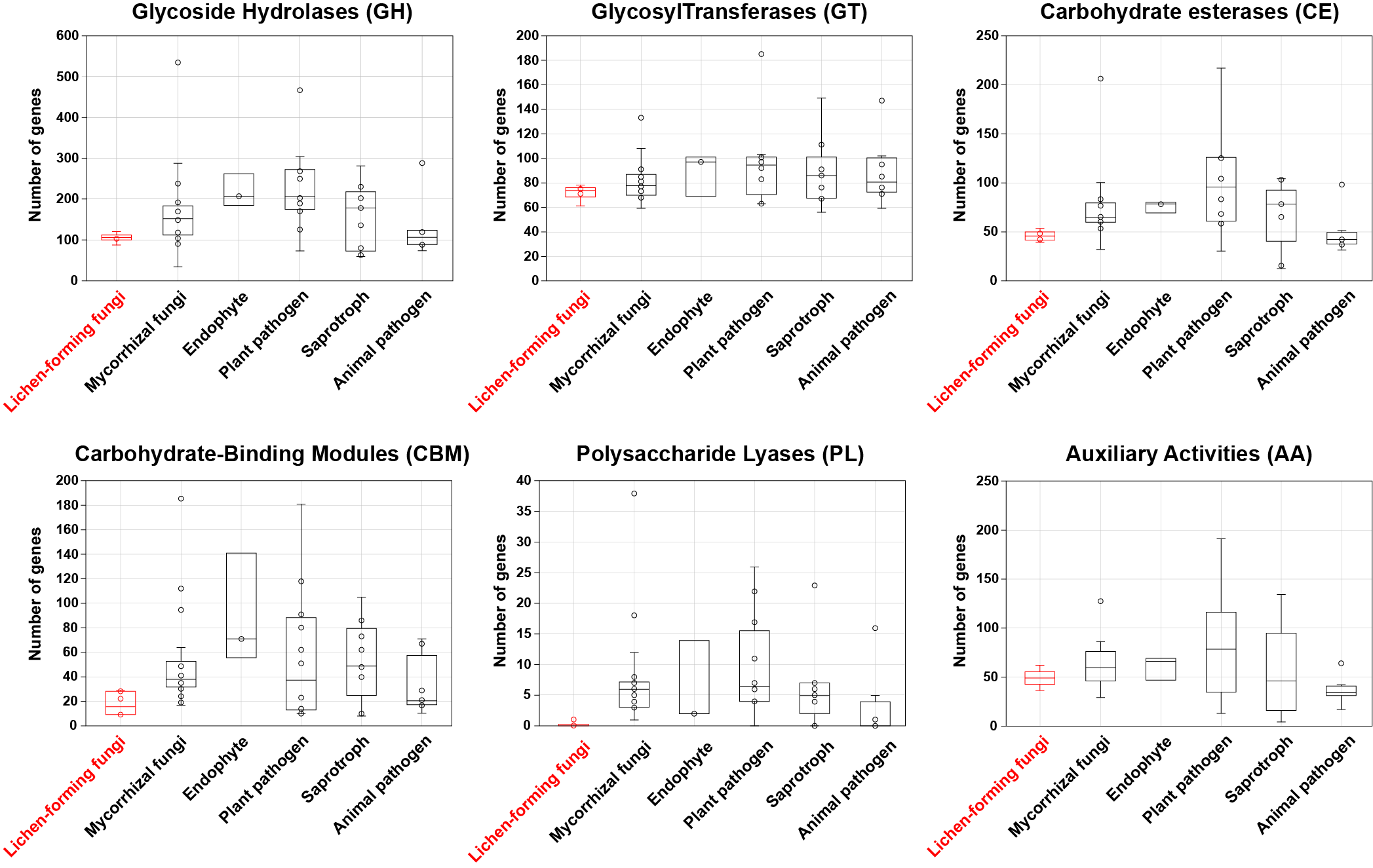


**Supplementary Fig. S3. Distribution of CAZyme genes among diverse fungal lifestyles**

Comparative analysis of CAZyme genes families among fungal lifestyles. In order from the top left glycoside hydrolases, glycosyltransferases, carbohydrate esterases, carbohydrate-binding modules, polysaccharide lyases, and auxiliary activities classes of CAZymes. The red box plot indicates lichen-forming fungi.

**
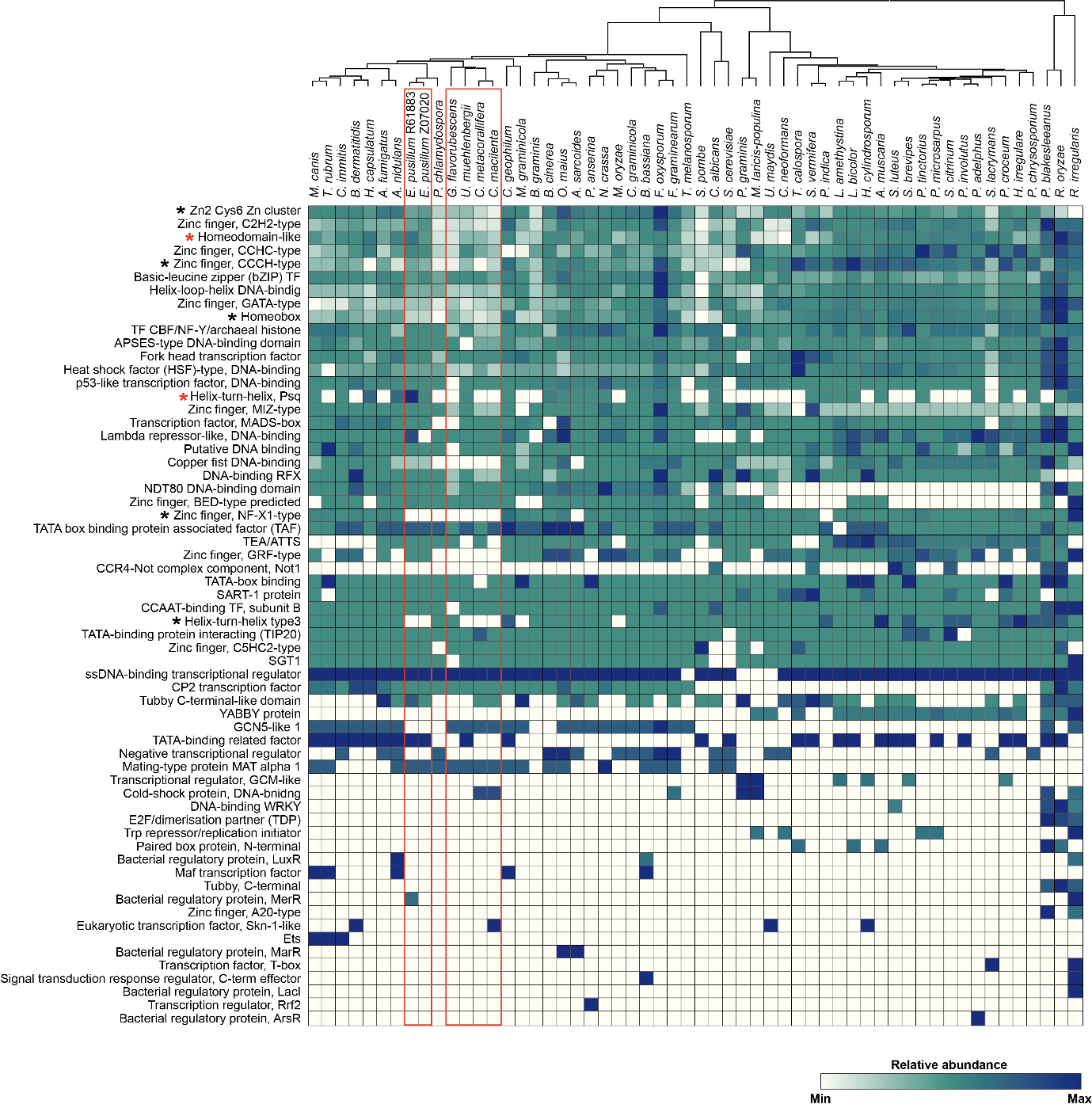
 Supplementary Fig. S4. Transcription factor (TF) gene families in 56 fungal species**

Total TF gene families are classified by DNA-binding domains, which are mostly found in fungal species (Shelest, 2017). The white color indicates the minimum number of genes and the blue color indicates the maximum number of genes. The part shown in the red line is the result of lichen-forming fungi. Black asterisk means massively contracted TF families in lichen-forming fungi, and the red asterisk indicates duplicated TF families only in *E. pusillum* R61883.


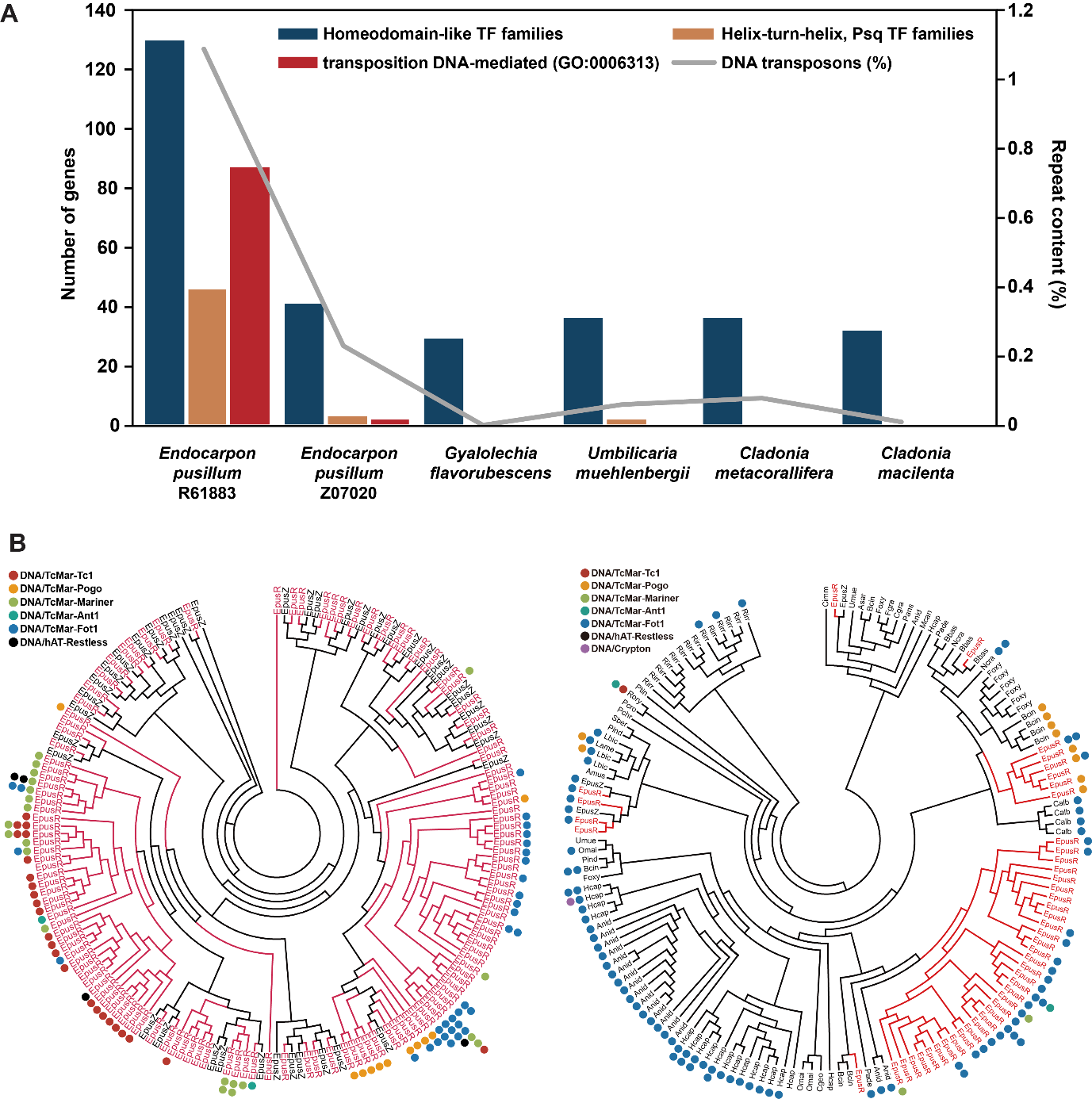


**Supplementary Fig. S5. Specific TF duplication in *E. pusillum* R61883 driven by repeat sequences.**

(A) Correlation among the expanded gene families, transposition DNA-mediated genes (GO:0006313), and DNA transposons. The bar graph represents the number of homeodomain-like, helix-turn-helix, psq, and transposition DNA-mediated genes, and the line graph represent the percentage of DNA transposons in each species. (B) Phylogenetic tree of homeodomain-like and helix-turn-helix, psq genes in *E. pusillum* isolates respectively (bootstrap value: 1000). Red color represents genes of *E. pusillum* R61883, which are significantly expanded. Colored circles indicate the number of DNA transposons within 3kb of TF genes and colors are a classification of DNA transposons.


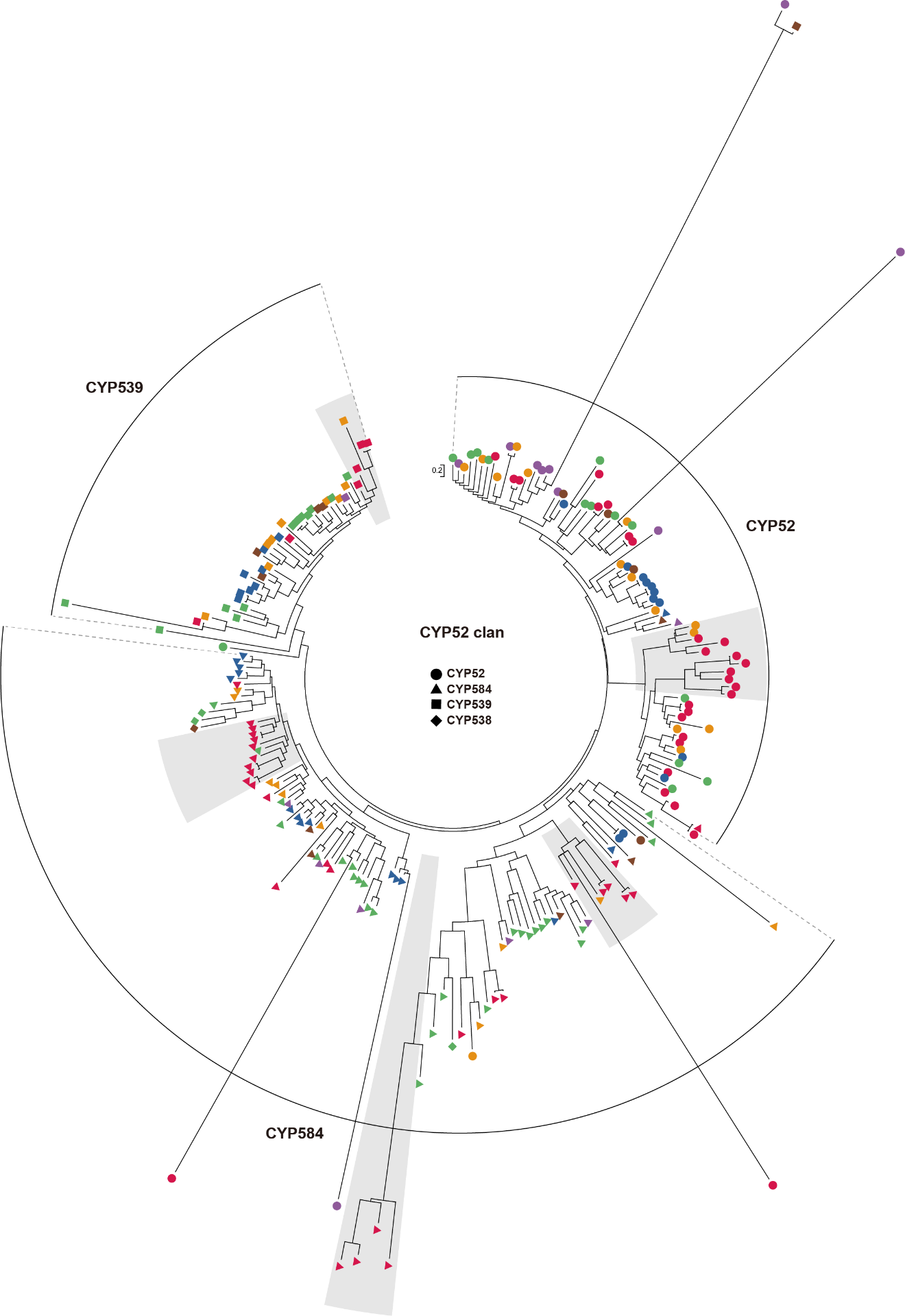

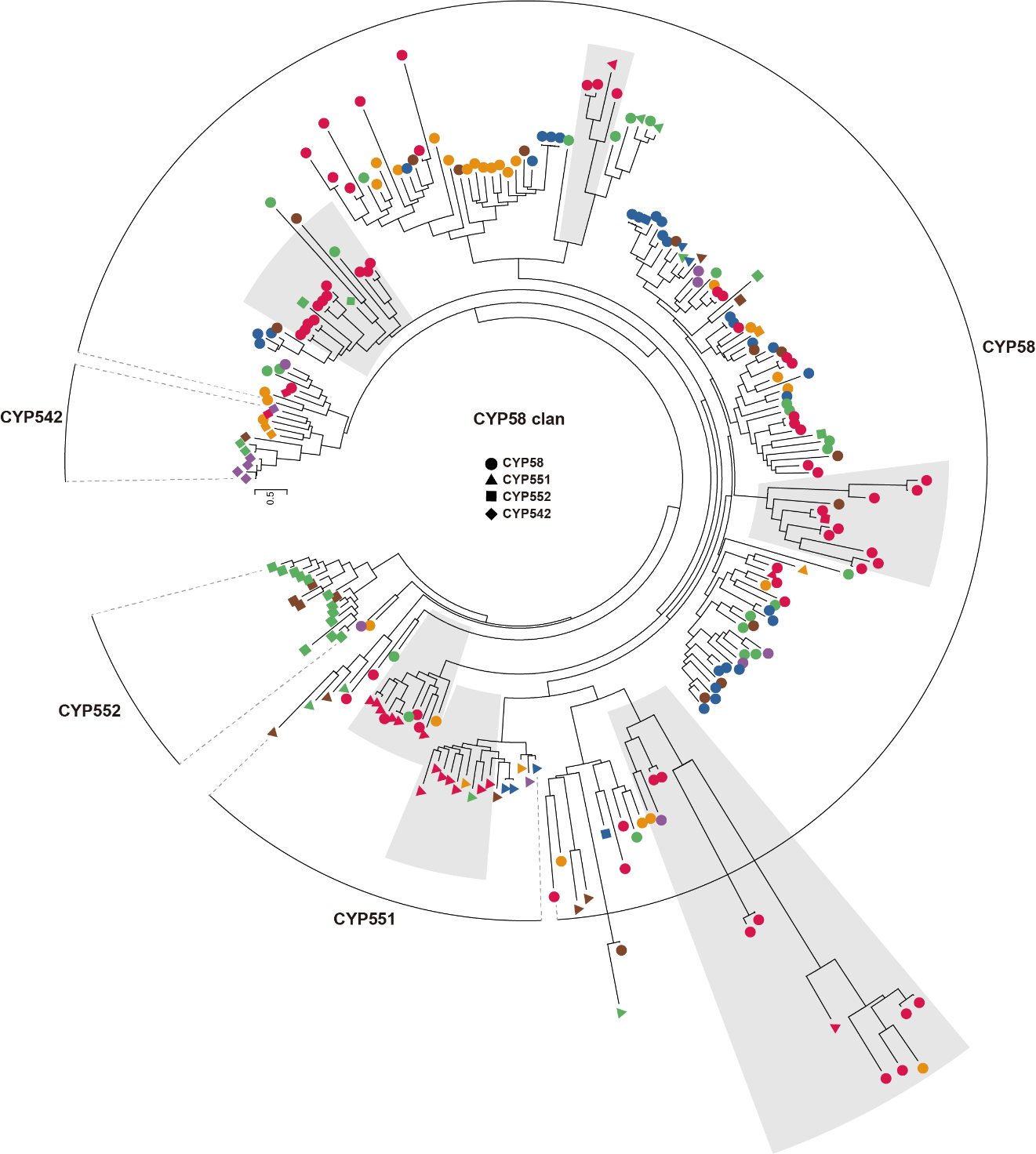


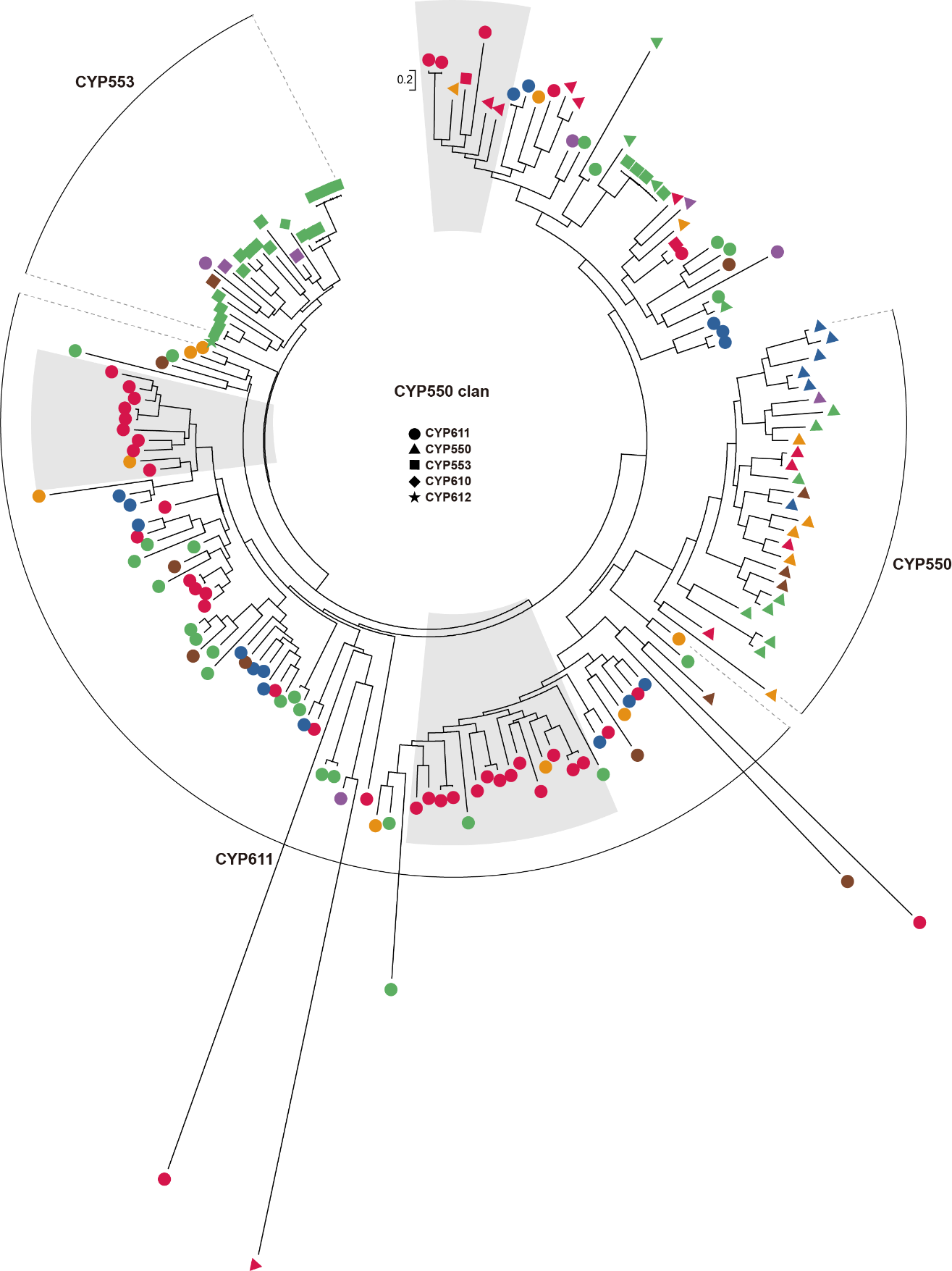


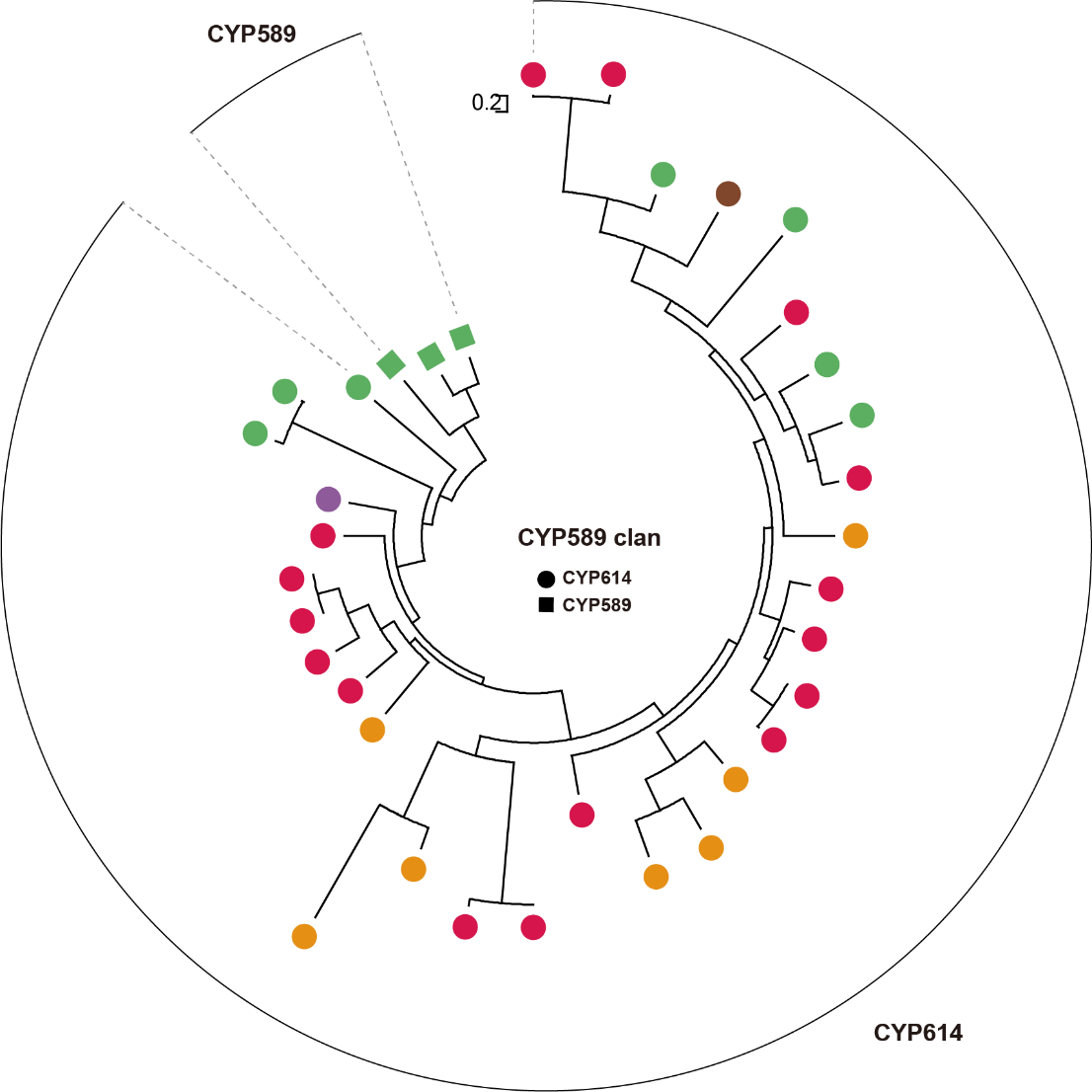


**Supplementary Fig. S6. Maximum-likelihood phylogenetic analysis of expanded cytochrome P450 families**

Phylogeny of each expanded cytochrome P450 families in ascomycetes fungi. Expanded families were shown with clans to which each family belongs. The maximum-likelihood phylogenetic tree was constructed by RAxML with 1000 bootstrap values. Families in each clan were classified with the shape of symbols. Colored marks mean each lifestyle of fungi. Red, orange, purple, green, brown, and blue indicate lichen-forming fungi, mycorrhizal fungi, endophyte, plant pathogen, saprotroph, and animal pathogen respectively. Gray shaded region shows lichen-forming fungi expanded groups or lichen-forming fungi-specific groups.
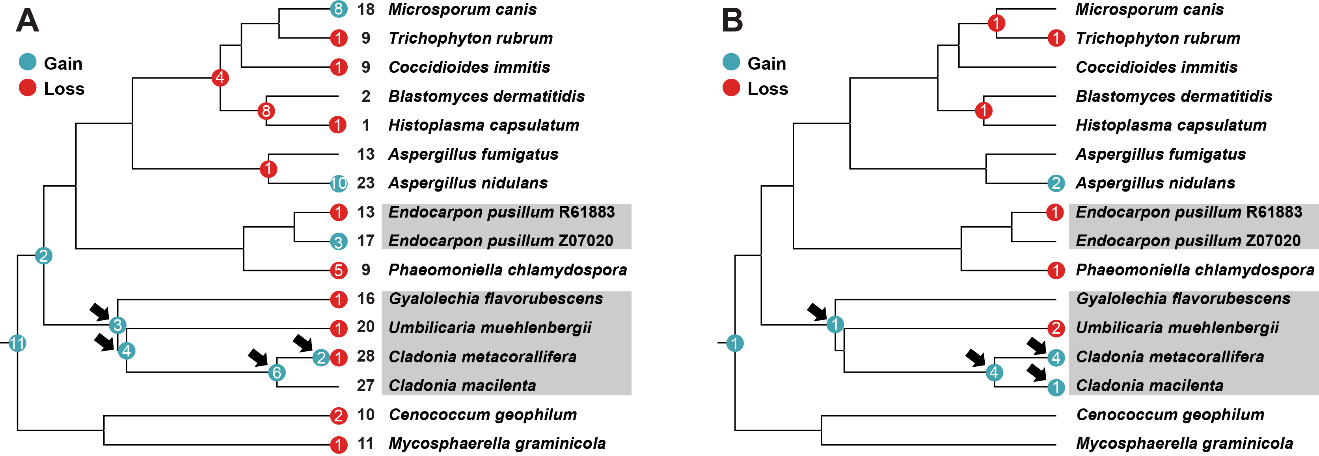


**Supplementary Fig. S7. Gain and loss of PKS and PKS-like genes in lichen-forming fungi**

Reconciliation analysis of PKS and PKS-like genes. The black arrow shows a gain of genes during the evolution of lichen-forming fungi. (A) Gain and loss of PKS genes in Eurotiomycetes, Lecanoromycetes, and Dothideomycetes. Blue circles indicate a gain of genes and a red circle means a loss of genes. (B) Changes in PKS-like genes during lichen-forming fungi evolution.


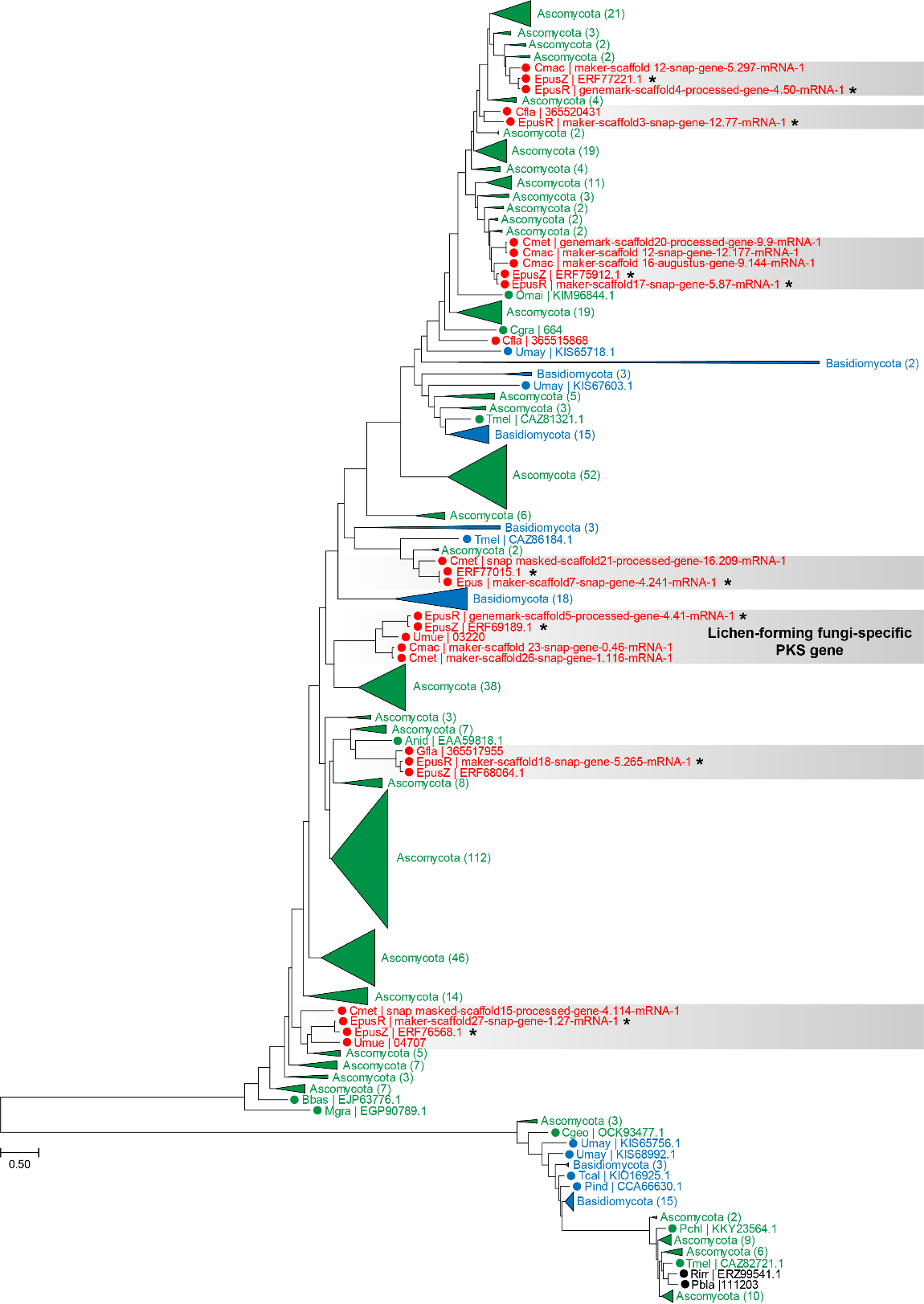


**Supplementary Fig. S8. Lichen-forming fungi unique polyketide synthase genes**

Polyketide synthase (PKS) genes in 56 fungal species. PKS gene of lichen-forming fungi represented by red color. Green color means PKS genes in Ascomycota, blue color is Basidiomycota and other PKS genes are black color. The numbers in the brackets are the number of PKS genes in each group. Gray shading indicates the PKS genes, which are shared with only *E. pusillum* isolates and Lecanoromycetes lichen-forming fungi. Genes of *E. pusillum* isolates were marked with an asterisk.


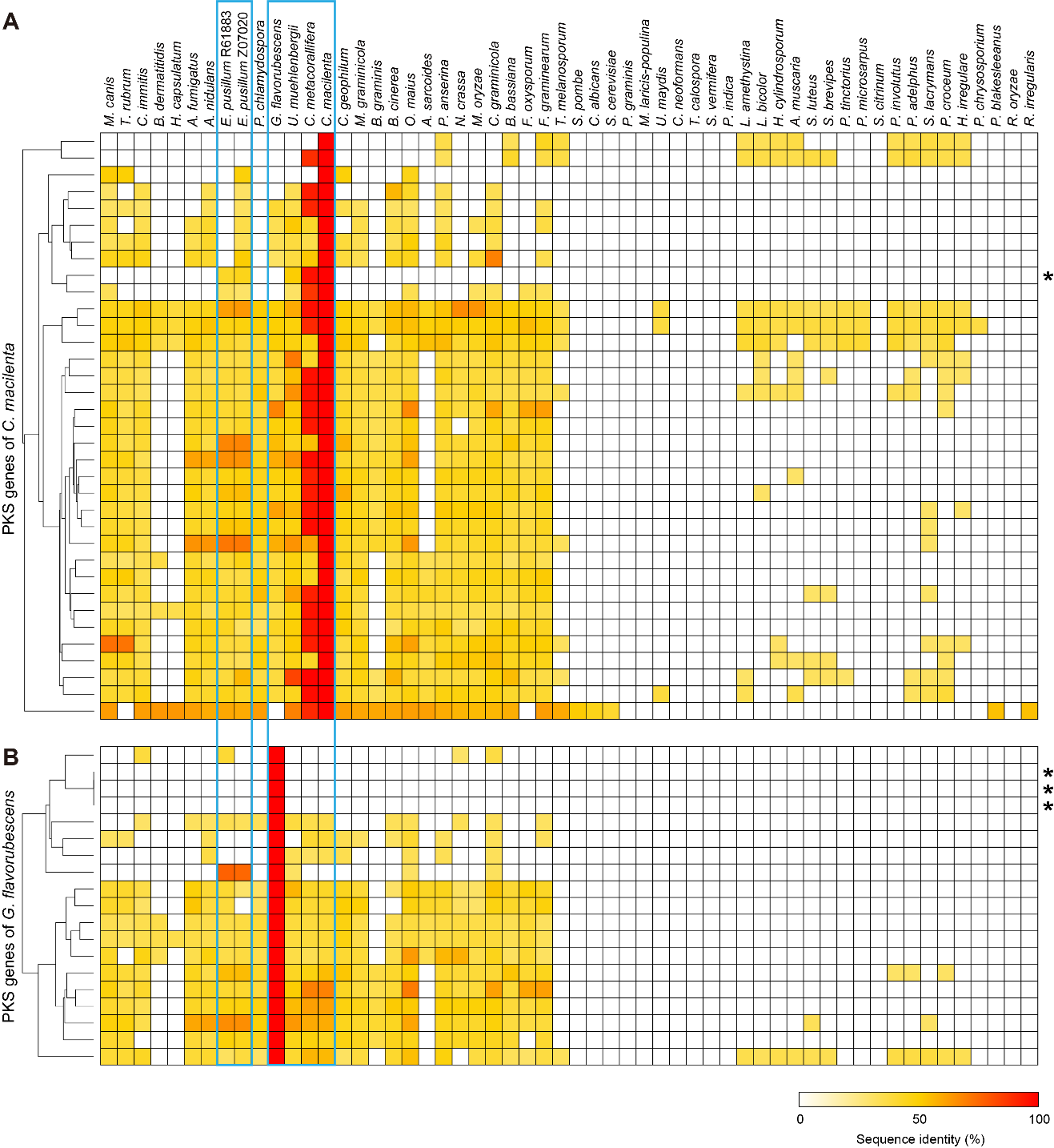


**Supplementary Fig. S9. Presence and absence of lichen-forming fungi PKS genes in 56 fungal species**

(A) BLAST search using PKS genes of *C. macilenta* as a reference. Red color means high sequence identity. The asterisk indicates lichen-specific PKS genes or species-specific PKS genes. (B) Sequence identity analysis of SSPs in *G. flavorubescens*.


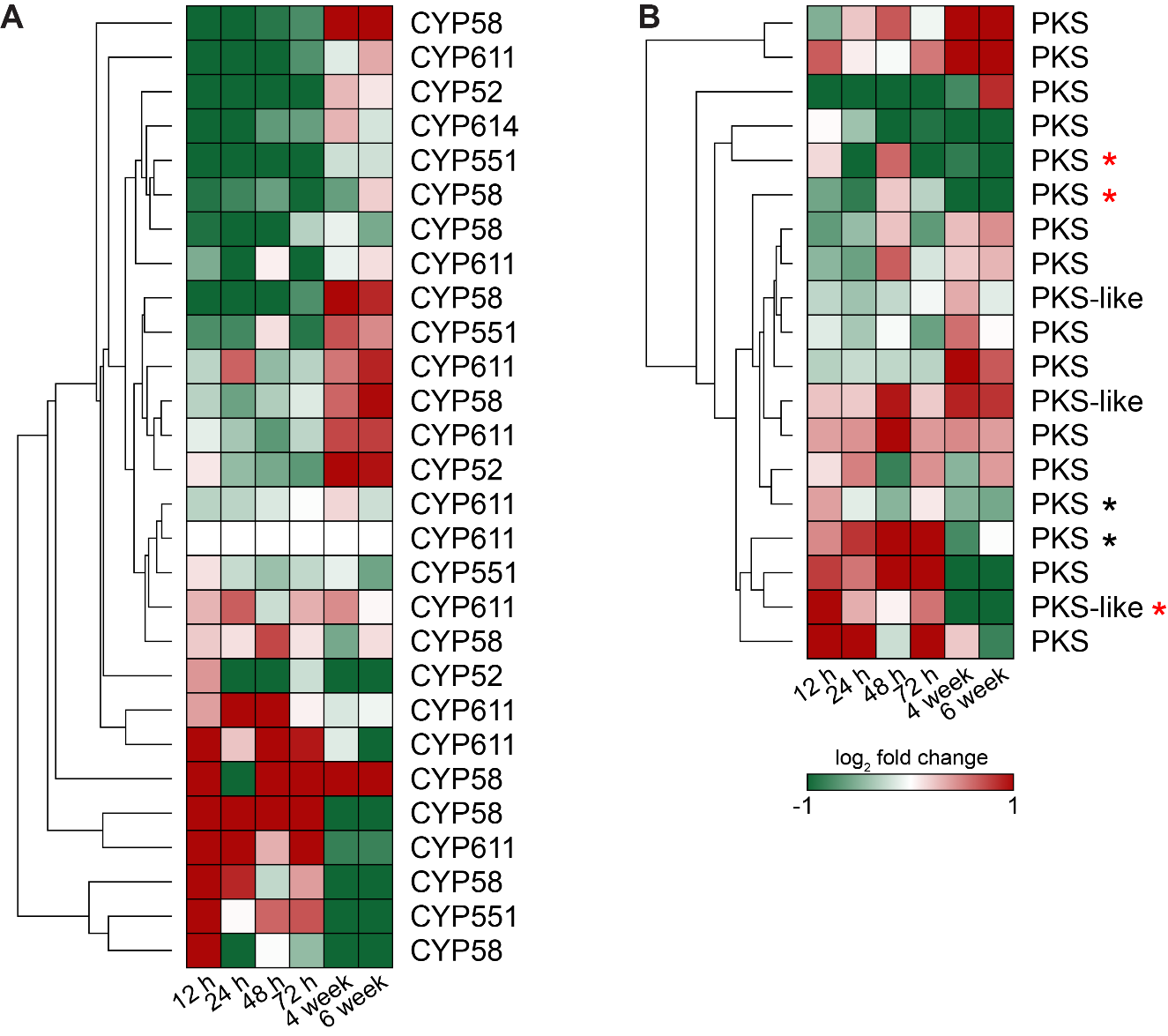


**Supplementary Fig. S10. Expression profile of cytochrome P450 and PKS genes in *G. flavorubescens***

Expression of (A) expanded CYP families and (B) PKS genes in symbiosis of *G. flavorubescens*. Each gene family was clustered using a hierarchical clustering method. (B) The red asterisk indicates the *G. flavorubescens*-specific PKS genes and the black asterisk means the *G. flavorubescens* PKS genes that shared with *Endocarpon* spp.


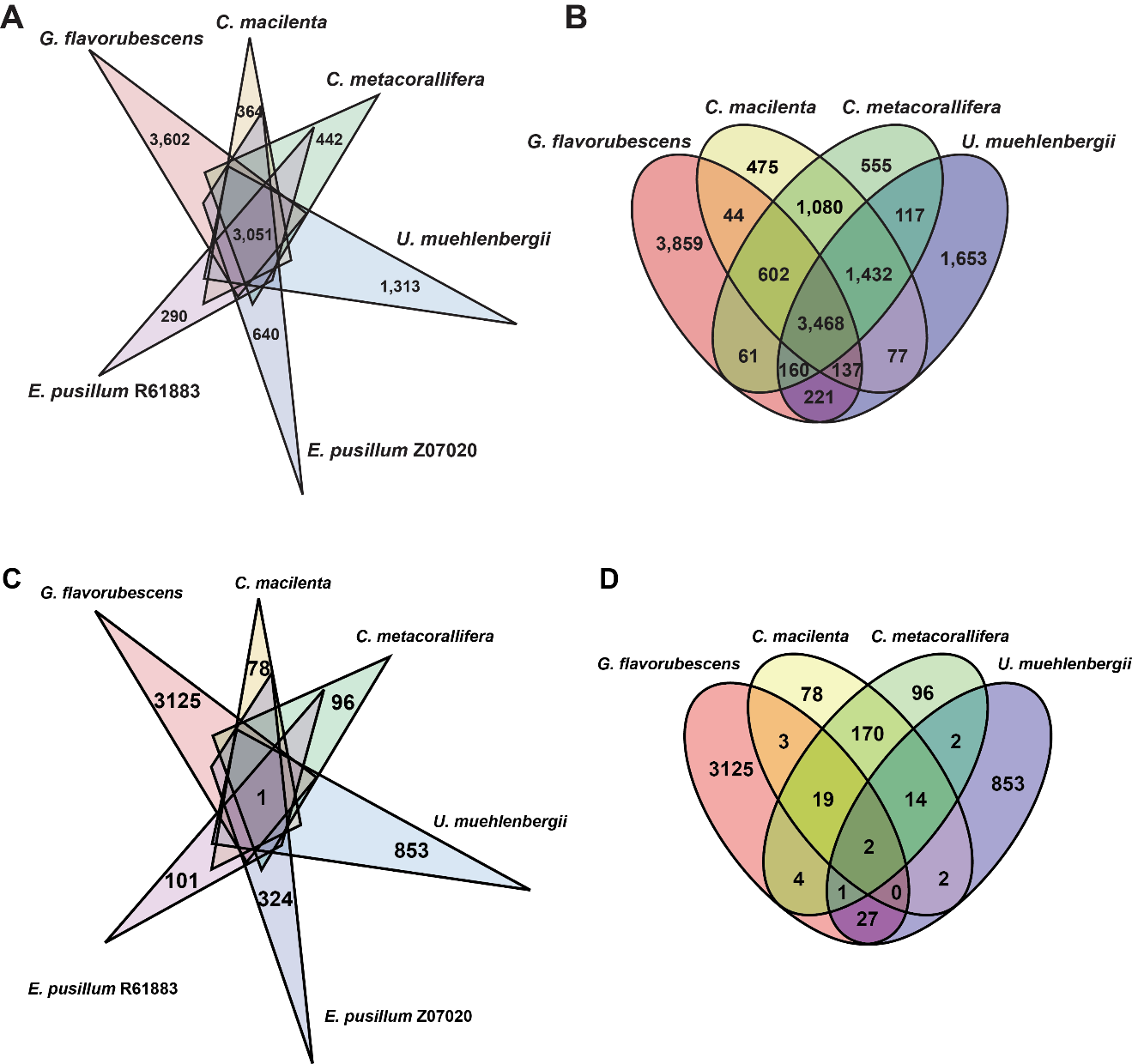


**Supplementary Fig. S11. Core and specific genes among lichen-forming fungi**

(A) The number of species-specific clusters and a core-cluster for the six lichen-forming fungi. The species-specific clusters are in each apex and the core-cluster is in the middle. (B) Ortholog clusters shared among the four Lecanoromycetes lichen-forming fungi. (C) and (D) Lichen-specific ortholog groups which are the result of ortholog clustering with 56 fungal species. Lichen-specific gene means that it is not present in the other non-lichen fungi.


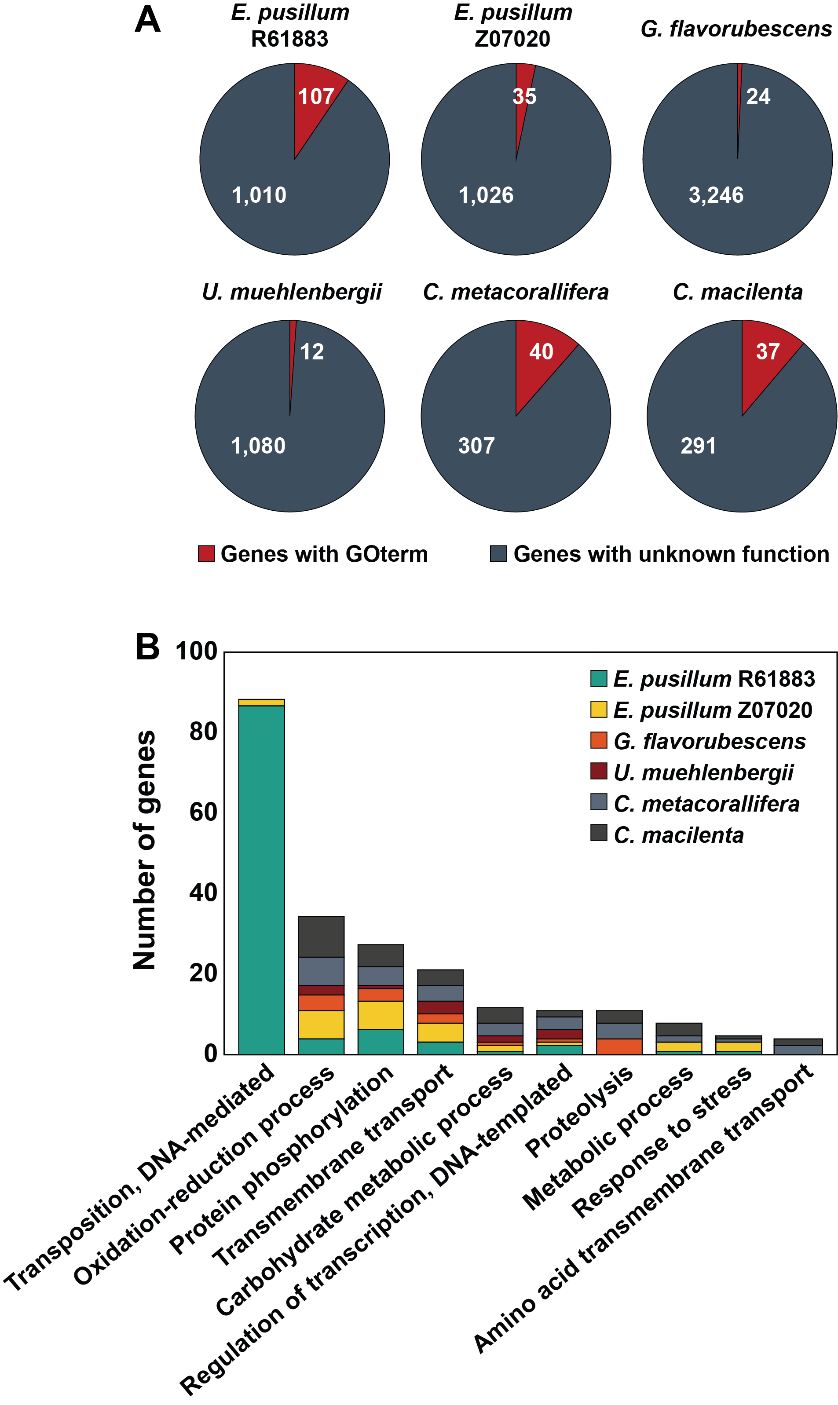


**Supplementary Fig. S12. Major functions of the lichen-specific genes**

The functions of lichen-specific genes were predicted by Gene Ontology (GO) term analysis. (A) The proportion of genes with GO terms in the lichen-specific genes. (B) The top 10 popular GO terms in the lichen-specific genes.


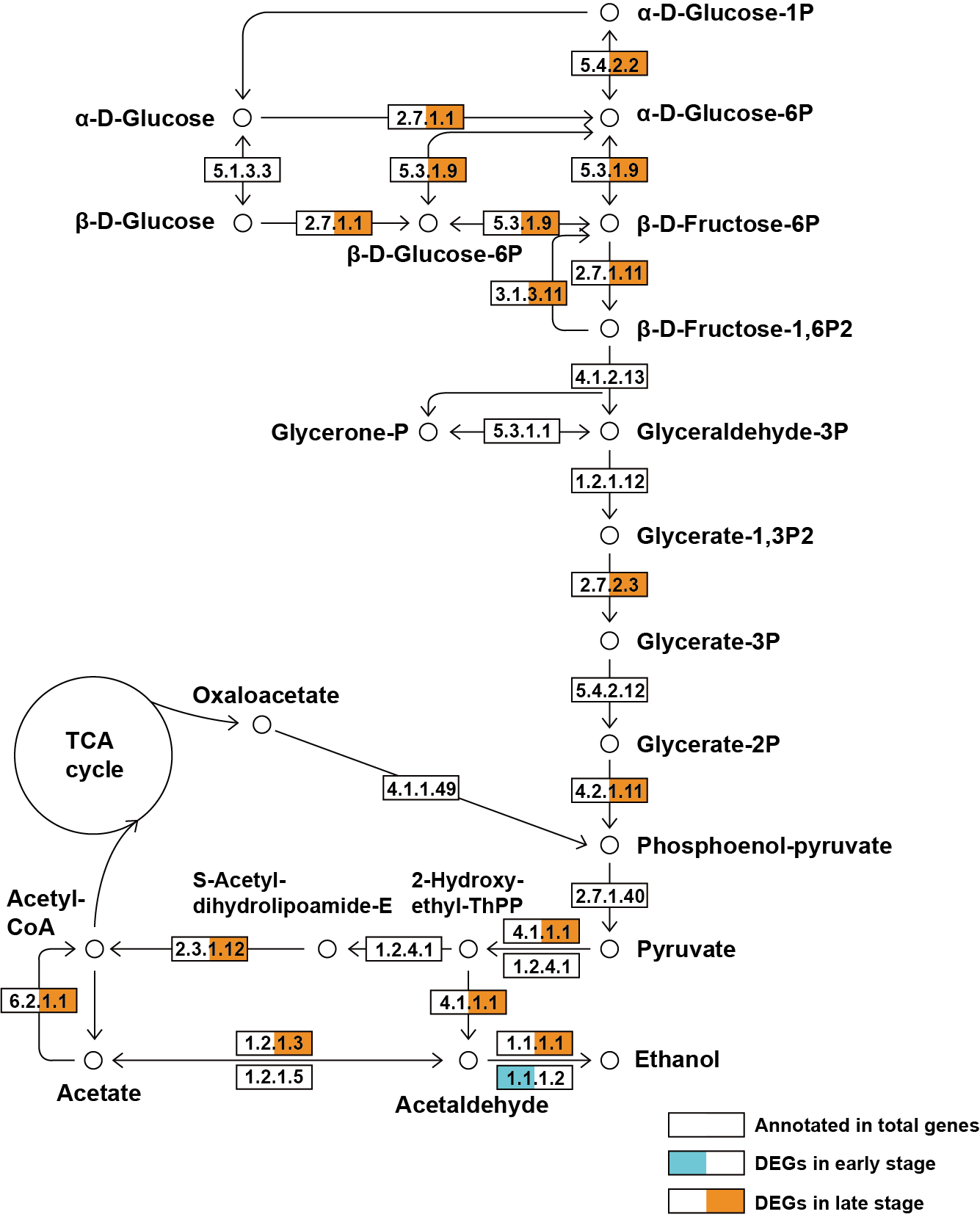


**Supplementary Fig. S13. Pathway analysis of glycolysis/gluconeogenesis in *G. flavorubescens***

The simplified schematic diagram of the glycolysis/gluconeogenesis pathway in the KEGG pathway. Only nodes with mapped genes of *G. flavorubescens* are displayed as white boxes. Nodes marked in blue are associated with differentially expressed genes (DEGs) in the early stage of *G. flavorubescens* resynthesis and nodes marked in orange are associated with DEGs in the late stage.


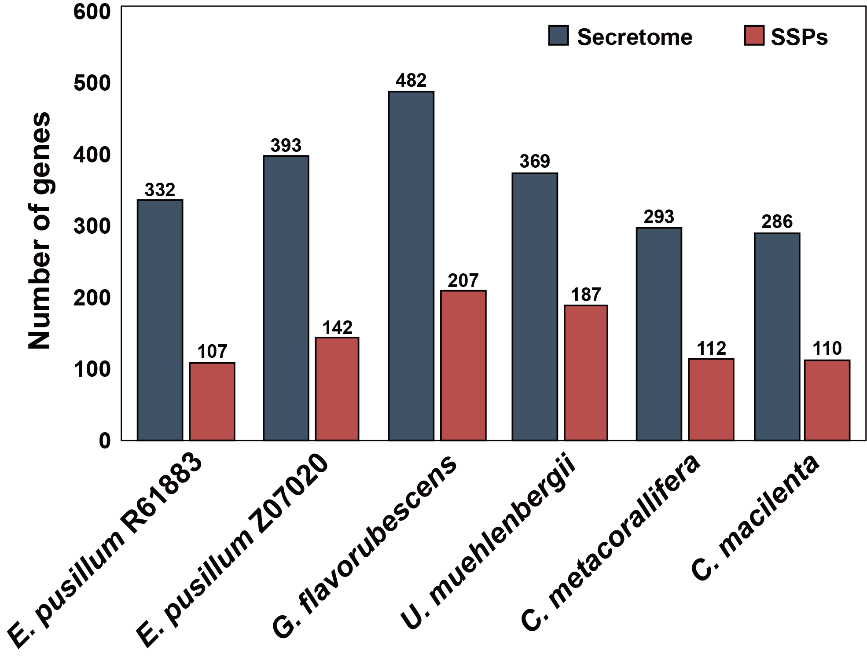


**Supplementary Fig. S14. Number of secreted proteins and SSPs in lichen-forming fungi**

Predicted secreted proteins and small secreted proteins (SSPs) in six lichen-forming fungi. The blue bar and red bar represent the number of secreted protein and SSPs respectively.


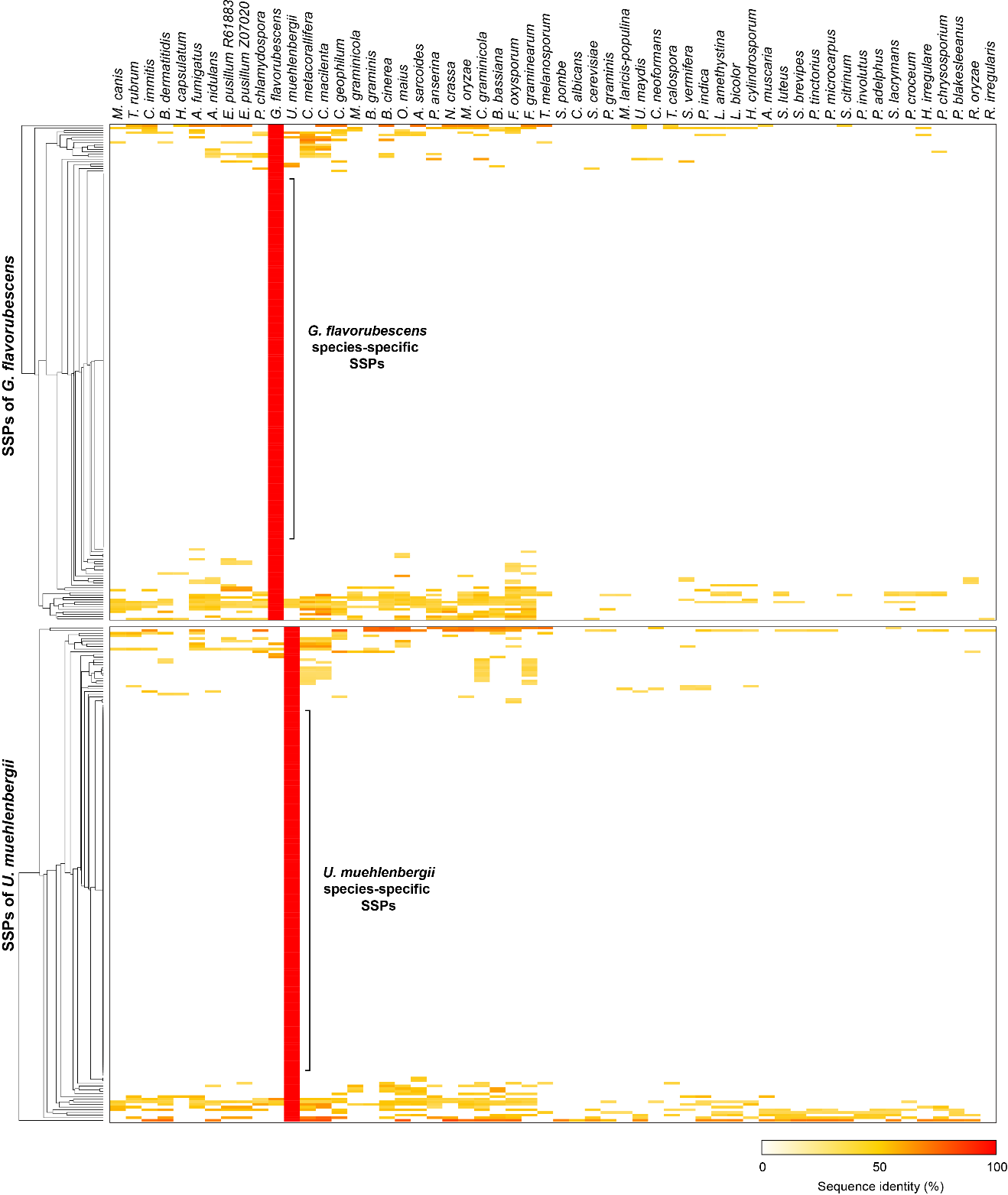


**Supplementary Fig. S15. Species-specific SSPs in *G. flavorubescens* and *U. muehlenbergii***

Sequence identity analysis with SSPs of 56 fungal species (BLAST E-value 1 × 10^−5^). SSPs of *G. flavorubescens* and *U. muehlenbergii* were used as a reference respectively, and almost all of the genes were species-specific.
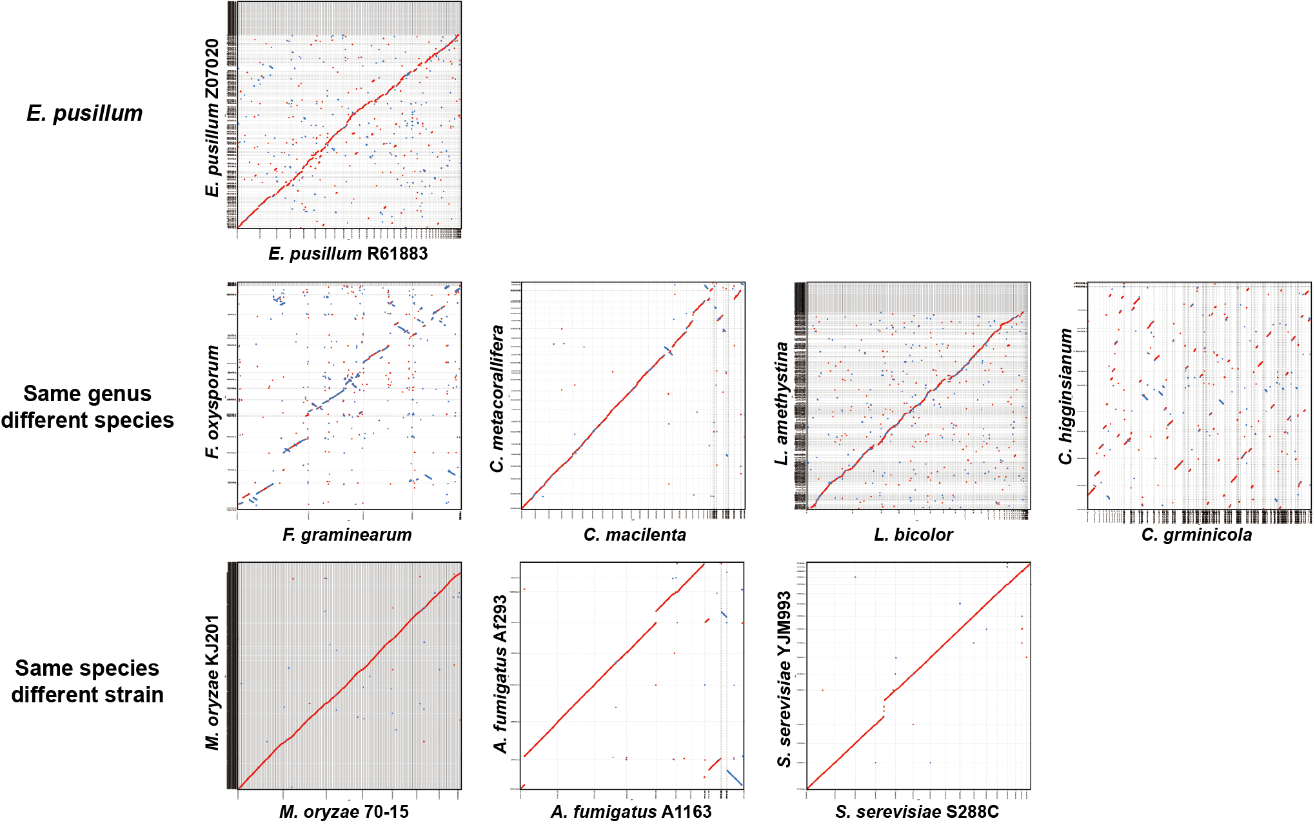


**Supplementary Fig. S16. Synteny analysis between *E. pusillum* isolates**

To delineate the two *E. pusillum* isolates Z07020 and R61883, dot plots were constructed using different species belong to the same genus and different strains belong to the same species of the selected fungal species. The *E. pusillum* dot plot is more similar to the dot plots of different species in the same genus.

**Supplementary Table S1. Conserved synteny regions between lichen-forming fungi**

|  | Overlapping Length (bp) | | Overlapping Ratio (%) | |
| --- | --- | --- | --- | --- |
|  | Query | Reference | Query | Reference |
| *C. macilenta* - *C. metacorallifera* | 24,397,165 | 24,403,396 | 65.7% | 66.5% |
| *C. macilenta* - *U. muehlenbergii* | 2,537,858 | 2,549,399 | 6.8% | 7.3% |
| *C. macilenta* - *G. flavorubescens* | 2,274,687 | 2,276,745 | 6.1% | 6.6% |
| *C. macilenta* - *E. pusillum R61883* | 1,306,448 | 1,300,376 | 3.5% | 3.5% |
| *C. macilenta - E. pusillum Z07020* | 1,277,251 | 1,272,538 | 3.4% | 3.4% |
| *E. pusillum R61883 - E. pusillum Z07020* | 18,978,499 | 18,989,224 | 50.8% | 51.1% |

**Supplementary Table S2. Predicted secondary metabolite synthase genes**

| **Species name** | **PKS** | **PKS-like** | **NRPS** | **NRPS-like** | **HYBRID** | **DMAT** | **Total** |
| --- | --- | --- | --- | --- | --- | --- | --- |
| *Microsporum canis* | 18 | 1 | 13 | 8 | 3 | 6 | 49 |
| *Trichophyton rubrum* | 9 | 0 | 12 | 1 | 1 | 3 | 26 |
| *Coccidioides immitis* | 9 | 2 | 5 | 3 | 1 | 1 | 21 |
| *Blastomyces dermatitidis* | 2 | 1 | 8 | 6 | 0 | 8 | 25 |
| *Histoplasma capsulatum* | 1 | 1 | 5 | 4 | 0 | 1 | 12 |
| *Aspergillus fumigatus* | 13 | 2 | 13 | 5 | 1 | 7 | 41 |
| *Aspergillus nidulans* | 23 | 4 | 11 | 12 | 1 | 5 | 56 |
| *Endocarpon pusillum R61883* | 13 | 1 | 2 | 3 | 0 | 3 | 22 |
| *Endocarpon pusillum Z07020* | 17 | 2 | 3 | 3 | 1 | 2 | 28 |
| *Phaeomoniella chlamydospora* | 9 | 1 | 5 | 2 | 0 | 0 | 17 |
| *Gyalolechia flavorubescens* | 16 | 3 | 1 | 7 | 2 | 2 | 31 |
| *Umbilicaria muehlenbergii* | 20 | 1 | 0 | 2 | 0 | 1 | 24 |
| *Cladonia metacorallifera* | 28 | 11 | 2 | 9 | 2 | 0 | 52 |
| *Cladonia macilenta* | 27 | 8 | 4 | 8 | 1 | 0 | 48 |
| *Cenococcum geophilum* | 10 | 2 | 7 | 3 | 1 | 2 | 25 |
| *Mycosphaerella graminicola* | 11 | 2 | 6 | 6 | 0 | 0 | 25 |
| *Blumeria graminis* | 1 | 1 | 1 | 1 | 0 | 0 | 4 |
| *Botrytis cinerea* | 16 | 6 | 6 | 8 | 0 | 1 | 37 |
| *Oidiodendron maius* | 44 | 2 | 9 | 10 | 2 | 3 | 70 |
| *Ascocoryne sarcoides* | 2 | 1 | 3 | 4 | 1 | 1 | 12 |
| *Podospora anserina* | 17 | 2 | 6 | 5 | 3 | 1 | 34 |
| *Neurospora crassa* | 8 | 1 | 3 | 2 | 0 | 1 | 15 |
| *Magnaporthe oryzae* | 24 | 2 | 8 | 6 | 5 | 3 | 48 |
| *Colletotrichum graminicola* | 37 | 1 | 5 | 7 | 4 | 6 | 60 |
| *Beauveria bassiana* | 12 | 1 | 13 | 7 | 3 | 0 | 36 |
| *Fusarium oxysporum* | 12 | 0 | 13 | 11 | 2 | 3 | 41 |
| *Fusarium graminearum* | 14 | 1 | 10 | 11 | 1 | 0 | 37 |
| *Tuber melanosporum* | 2 | 1 | 1 | 2 | 0 | 0 | 6 |
| *Schizosaccharomyces pombe* | 0 | 1 | 1 | 1 | 0 | 0 | 3 |
| *Candida albicans* | 0 | 2 | 0 | 0 | 0 | 0 | 2 |
| *Saccharomyces cerevisiae* | 0 | 1 | 0 | 0 | 0 | 0 | 1 |
| *Puccinia graminis* | 2 | 0 | 0 | 1 | 0 | 0 | 3 |
| *Melampsora laricis-populina* | 0 | 1 | 0 | 2 | 0 | 0 | 3 |
| *Ustilago maydis* | 3 | 2 | 3 | 6 | 0 | 2 | 16 |
| *Cryptococcus neoformans* | 0 | 0 | 0 | 1 | 0 | 0 | 1 |
| *Tulasnella calospora* | 1 | 0 | 0 | 1 | 0 | 0 | 2 |
| *Sebacina vermifera* | 0 | 0 | 0 | 0 | 0 | 0 | 0 |
| *Piriformospora indica* | 1 | 0 | 0 | 1 | 0 | 1 | 3 |
| *Laccaria amethystina* | 3 | 0 | 2 | 1 | 0 | 1 | 7 |
| *Laccaria bicolor* | 3 | 1 | 0 | 2 | 0 | 1 | 7 |
| *Hebeloma cylindrosporum* | 3 | 1 | 1 | 4 | 0 | 1 | 10 |
| *Amanita muscaria* | 3 | 0 | 0 | 1 | 0 | 1 | 5 |
| *Suillus luteus* | 3 | 0 | 1 | 4 | 0 | 0 | 8 |
| *Suillus brevipes* | 3 | 0 | 1 | 5 | 0 | 0 | 9 |
| *Pisolithus tinctorius* | 2 | 0 | 1 | 2 | 0 | 0 | 5 |
| *Pisolithus microcarpus* | 2 | 0 | 1 | 4 | 0 | 0 | 7 |
| *Scleroderma citrinum* | 1 | 2 | 0 | 4 | 1 | 0 | 8 |
| *Paxillus involutus* | 4 | 2 | 3 | 9 | 2 | 0 | 20 |
| *Paxillus adelphus* | 2 | 0 | 1 | 2 | 2 | 0 | 7 |
| *Serpula lacrymans* | 6 | 0 | 8 | 6 | 2 | 3 | 25 |
| *Piloderma croceum* | 6 | 1 | 1 | 8 | 0 | 3 | 19 |
| *Heterobasidion irregulare* | 3 | 2 | 0 | 8 | 0 | 1 | 14 |
| *Phanerochaete chrysosporium* | 2 | 0 | 1 | 15 | 0 | 0 | 18 |
| *Phycomyces blakesleeanus* | 0 | 1 | 0 | 4 | 0 | 0 | 5 |
| *Rhizopus oryzae* | 0 | 0 | 0 | 6 | 0 | 0 | 6 |
| *Rhizophagus irregularis* | 0 | 1 | 0 | 0 | 0 | 0 | 1 |

**Supplementary Table S3. Summary of ortholog clustering with lichen-forming fungi**

| **Fungal species** | **Orthogroups** | **Categories** | **Number of clusters** | **Number of genes** |
| --- | --- | --- | --- | --- |
| Lichen-forming fungi (6 species) | Pan clusters |  | 15,712 | 54,282 |
|  | Core clusters | Total | 3,051 | 23,234 |
|  |  | *E. pusillum* R61883 core gene | - | 4,130 |
|  |  | *E. pusillum* Z07020 core gene | - | 3,761 |
|  |  | *G. flavorubescens* core gene | - | 3,735 |
|  |  | *U. muehlenbergii* core gene | - | 3,626 |
|  |  | *C. metacorallifera* core gene | - | 4,025 |
|  |  | *C. macilenta* core gene | - | 3,957 |
|  | Lineage-specific clusters | *Endocarpon* lineage-specific clusters | 1,220 | 2,970 |
|  |  | *Cladonia* lineage-specific clusters | 739 | 1,586 |
|  | Orphan genes | *E. pusillum* R61883 orphan gene | 290 | 324 |
|  |  | *E. pusillum* Z07020 orphan gene | 640 | 650 |
|  |  | *G. flavorubescens* orphan gene | 3,602 | 3,624 |
|  |  | *U. muehlenbergii* orphan gene | 1,313 | 1,340 |
|  |  | *C. metacorallifera* orphan gene | 442 | 447 |
|  |  | *C. macilenta* orphan gene | 364 | 364 |
| Lecanoromycetes (4 species) | Pan clusters |  | 13,941 | 35,792 |
|  | Core clusters | Total | 3,468 | 17,365 |
|  |  | *G. flavorubescens* core gene | - | 4,226 |
|  |  | *U. muehlenbergii* core gene | - | 4,138 |
|  |  | *C. metacorallifera* core gene | - | 4,540 |
|  |  | *C. macilenta* core gene | - | 4,461 |
|  | Lineage-specific clusters | *Cladonia* lineage-specific clusters | 1,080 | 2,358 |
|  | Orphan genes | *G. flavorubescens* orphan gene | 3,859 | 3,887 |
|  |  | *U. muehlenbergii* orphan gene | 1,653 | 1,667 |
|  |  | *C. metacorallifera* orphan gene | 555 | 562 |
|  |  | *C. macilenta* orphan gene | 475 | 475 |

**Supplementary Table S4. Go term enrichment test with differentially expressed conserved genes in *G. flavorubescens***

| **GO IDs** | **Terms** | **Annotated** | **Observed** | **Expected** | **Significance** |
| --- | --- | --- | --- | --- | --- |
| <Up-regulated in early stage> | | | | | |
| GO:0051276 | chromosome organization | 40 | 10 | 4.01 | 0.0048 |
| GO:0006281 | DNA repair | 68 | 14 | 6.83 | 0.0062 |
| GO:0009147 | pyrimidine nucleoside triphosphate metabolic process | 2 | 2 | 0.2 | 0.01 |
| GO:0018193 | peptidyl-amino acid modification | 28 | 7 | 2.81 | 0.0176 |
| GO:0006631 | fatty acid metabolic process | 12 | 4 | 1.2 | 0.0256 |
| GO:0043543 | protein acylation | 3 | 2 | 0.3 | 0.0281 |
| GO:0006457 | protein folding | 38 | 8 | 3.81 | 0.0313 |
| GO:0006464 | cellular protein modification process | 215 | 30 | 21.58 | 0.0338 |
| GO:0016570 | histone modification | 13 | 4 | 1.3 | 0.0342 |
| <Down-regulated in early stage> | | | | | |
| GO:0006355 | regulation of transcription, DNA-templated | 126 | 39 | 21.67 | 0.00007 |
| GO:0055085 | transmembrane transport | 272 | 62 | 46.79 | 0.0073 |
| GO:0042157 | lipoprotein metabolic process | 10 | 5 | 1.72 | 0.0174 |
| GO:0042158 | lipoprotein biosynthetic process | 10 | 5 | 1.72 | 0.0174 |
| GO:1903509 | liposaccharide metabolic process | 10 | 5 | 1.72 | 0.0174 |
| GO:0055114 | oxidation-reduction process | 440 | 90 | 75.69 | 0.0283 |
| GO:0044550 | secondary metabolite biosynthetic process | 2 | 2 | 0.34 | 0.0295 |
| GO:0044036 | cell wall macromolecule metabolic process | 2 | 2 | 0.34 | 0.0295 |
| GO:0007034 | vacuolar transport | 8 | 4 | 1.38 | 0.0339 |
| GO:0001932 | regulation of protein phosphorylation | 5 | 3 | 0.86 | 0.0385 |
| GO:0051338 | regulation of transferase activity | 5 | 3 | 0.86 | 0.0385 |
| GO:0071554 | cell wall organization or biogenesis | 5 | 3 | 0.86 | 0.0385 |
| <Up-regulated in late stage> | | | | | |
| GO:0006096 | glycolytic process | 8 | 6 | 1.79 | 0.0023 |
| GO:0009070 | serine family amino acid biosynthetic process | 4 | 4 | 0.89 | 0.0025 |
| GO:0042157 | lipoprotein metabolic process | 10 | 6 | 2.24 | 0.0111 |
| GO:0042158 | lipoprotein biosynthetic process | 10 | 6 | 2.24 | 0.0111 |
| GO:1903509 | liposaccharide metabolic process | 10 | 6 | 2.24 | 0.0111 |
| GO:0070646 | protein modification by small protein removal | 13 | 7 | 2.91 | 0.013 |
| GO:0006351 | transcription, DNA-templated | 181 | 53 | 40.48 | 0.0146 |
| GO:0051186 | cofactor metabolic process | 65 | 26 | 14.54 | 0.0168 |
| GO:0065009 | regulation of molecular function | 14 | 7 | 3.13 | 0.021 |
| GO:0051336 | regulation of hydrolase activity | 6 | 4 | 1.34 | 0.0252 |
| GO:0019438 | aromatic compound biosynthetic process | 274 | 78 | 61.28 | 0.0272 |
| GO:0018130 | heterocycle biosynthetic process | 287 | 81 | 64.19 | 0.0285 |
| GO:1901362 | organic cyclic compound biosynthetic process | 298 | 83 | 66.65 | 0.0352 |
| GO:0042401 | cellular biogenic amine biosynthetic process | 4 | 3 | 0.89 | 0.0371 |
| GO:0006796 | phosphate-containing compound metabolic process | 233 | 67 | 52.11 | 0.0394 |
| GO:0070647 | protein modification by small protein conjugation or removal | 19 | 8 | 4.25 | 0.0427 |
| GO:0006793 | phosphorus metabolic process | 234 | 67 | 52.34 | 0.0429 |
| GO:0035556 | intracellular signal transduction | 58 | 19 | 12.97 | 0.043 |
| GO:0019219 | regulation of nucleobase-containing compound metabolic process | 128 | 37 | 28.63 | 0.0459 |
| <Down-regulated in late stage> | | | | | |
| GO:0006189 | 'de novo' IMP biosynthetic process | 2 | 2 | 0.16 | 0.0065 |
| GO:0015703 | chromate transport | 2 | 2 | 0.16 | 0.0065 |
| GO:0061024 | membrane organization | 6 | 3 | 0.48 | 0.0087 |
| GO:0071166 | ribonucleoprotein complex localization | 3 | 2 | 0.24 | 0.0185 |
| GO:0015931 | nucleobase-containing compound transport | 3 | 2 | 0.24 | 0.0185 |
| GO:0051236 | establishment of RNA localization | 3 | 2 | 0.24 | 0.0185 |
| GO:0006611 | protein export from nucleus | 3 | 2 | 0.24 | 0.0185 |
| GO:0006403 | RNA localization | 3 | 2 | 0.24 | 0.0185 |
| GO:0051189 | prosthetic group metabolic process | 4 | 2 | 0.32 | 0.0349 |
| GO:0006694 | steroid biosynthetic process | 4 | 2 | 0.32 | 0.0349 |
| GO:0006414 | translational elongation | 10 | 3 | 0.81 | 0.0408 |

**Supplementary Table S5. Alignment with conserved genes between *E. pusillum* isolates**

|  | **Query** | **Reference** | **ACT1** | **TEF1** | **TUB1** | **TUB2** |
| --- | --- | --- | --- | --- | --- | --- |
| *E. pusillum* | *E. pusillum* Z07020 | *E. pusillum* R61883 | 100% | 99.6% | 100% | 99.5% |
| Same genus, different species | *F. graminearum* | *F. oxysporum* | 100% | 94.1% | 97.7% | 99.5% |
|  | *C. macilenta* | *C. metacorallifera* | 100% | 100% | 100% | 100% |
|  | *L. amethystina* | *L. bicolor* | 99.7% | 98.5% | 100% | 99.5% |
|  | *C. graminicola* | *C. higginsianum* | 100% | 97.6% | 97.5% | 99.5% |
| Same species, different strain | *M. oryzae* 70-15 | *M. oryzae* KJ201 | 100% | 100% | 100% | 100% |
|  | *A. fumigatus* A1163 | *A. fumigatus* Af293 | 100% | 100% | 100% | 100% |
|  | *S.cerevisiae* S288C | *S. cerevisiae* YJM993 | 100% | 100% | 100% | 99.7% |

**Supplementary Table S6. Synteny analysis of *E. pusillum* isolates**

|  | **Reference species - Query species** | **Length (bp)** | | **Ratio (%)** | |
| --- | --- | --- | --- | --- | --- |
|  |  | **Query** | **Reference** | **Query** | **Reference** |
| *E. pusillum* | *E. pusillum R61883 - E. pusillum Z07020* | 18,978,499 | 18,989,224 | 51% | 51% |
| Same genus different species | *F. graminearum - F. oxysporum* | 11,510,880 | 11,502,471 | 31% | 19% |
|  | *C. macilenta - C. metacorallifera* | 24,397,165 | 24,403,396 | 66% | 67% |
|  | *L. bicolor - L. amethystina* | 15,635,185 | 15,640,216 | 26% | 30% |
|  | *C. graminicola - C. higginsianum* | 12,770,527 | 12,834,010 | 25% | 25% |
| Same species different strain | *M. oryzae 70-15 - M. oryzae KJ201* | 41,114,602 | 41,109,952 | 100% | 115% |
|  | *A. fumigatus A1163 - A. fumigatus Af293* | 28,533,389 | 28,532,000 | 98% | 97% |
|  | *S. cerevisiae S288C - S. cerevisiae YJM993* | 11,838,757 | 11,840,737 | 98% | 95% |

**References**

1 Park, S. Y. *et al.* Draft genome sequence of *Endocarpon pusillum* strain KoLRILF000583. *Genome Announc.* **2**, doi:10.1128/genomeA.00452-14 (2014).

2 Wang, Y. Y. *et al.* Genome characteristics reveal the impact of lichenization on lichen-forming fungus Endocarpon pusillum Hedwig (Verrucariales, Ascomycota). *Bmc Genom.* **15**, 34, doi:10.1186/1471-2164-15-34 (2014).

3 Boluda, C. *et al.* Evaluating methodologies for species delimitation: the mismatch between phenotypes and genotypes in lichenized fungi (Bryoria sect. Implexae, Parmeliaceae). *Persoonia* **42**, 75-100 (2019).

4 Lumbsch, H. T. & Leavitt, S. D. Goodbye morphology? A paradigm shift in the delimitation of species in lichenized fungi. *Fungal Divers.* **50**, 59-72, doi:10.1007/s13225-011-0123-z (2011).

5 Feschotte, C. & Pritham, E. J. DNA transposons and the evolution of eukaryotic genomes. *Annu. Rev. Genet.* **41**, 331-368 (2007).

6 Rose, M. R. & Doolittle, W. F. Molecular biological mechanisms of speciation. *Science* **220**, 157-162, doi:10.1126/science.220.4593.157 (1983).
